# Mathematical modelling of spatio-temporal glioma evolution

**DOI:** 10.1186/1742-4682-10-47

**Published:** 2013-07-24

**Authors:** Maria Papadogiorgaki, Panagiotis Koliou, Xenofon Kotsiakis, Michalis E Zervakis

**Affiliations:** 1 Digital Image and Signal Processing Laboratory, Electronic and Computer Engineering Department, Technical University of Crete, Polytechnioupolis, Kounopidiana Campus, Chania, Crete 73100, Greece; 2 Department of Oncology, General Hospital of Chania “Agios Georgios”, Mournies, Chania, Crete 73300, Greece; 3 Department of Neurosurgery, General Hospital of Chania “Agios Georgios”, Mournies, Chania, Crete 73300, Greece

**Keywords:** Glioma, modelling, Spatio-temporal evolution, Cancer prediction, Tumor cells, Nutrients models, Proliferative, Hypoxic, Hypoglycemic, Necrotic regions

## Abstract

**Background:**

Gliomas are the most common types of brain cancer, well known for their aggressive proliferation and the invasive behavior leading to a high mortality rate. Several mathematical models have been developed for identifying the interactions between glioma cells and tissue microenvironment, which play an important role in the mechanism of the tumor formation and progression.

**Methods:**

Building and expanding on existing approaches, this paper develops a continuous three-dimensional model of avascular glioma spatio-temporal evolution. The proposed spherical model incorporates the interactions between the populations of four different glioma cell phenotypes (proliferative, hypoxic, hypoglychemic and necrotic) and their tissue microenvironment, in order to investigate how they affect tumor growth and invasion in an isotropic and homogeneous medium. The model includes two key variables involved in the proliferation and invasion processes of cancer cells; i.e. the extracellular matrix and the matrix-degradative enzymes concentrations inside the tumor and its surroundings. Additionally, the proposed model focuses on innovative features, such as the separate and independent impact of two vital nutrients, namely oxygen and glucose, in tumor growth, leading to the formation of cell populations with different metabolic profiles. The model implementation takes under consideration the variations of particular factors, such as the local cell proliferation rate, the variable conversion rates of cells from one category to another and the nutrient-dependent thresholds of conversion. All model variables (cell densities, ingredients concentrations) are continuous and described by reaction-diffusion equations.

**Results:**

Several simulations were performed using combinations of growth and invasion rates, for different evolution times. The model results were evaluated by medical experts and validated on experimental glioma models available in the literature, revealing high agreement between simulated and experimental results.

**Conclusions:**

Based on the experimental validation, as well as the evaluation by clinical experts, the proposed model may provide an essential tool for the patient-specific simulation of different tumor evolution scenarios and reliable prognosis of glioma spatio-temporal progression.

## Introduction and background

Cancer is the second most fatal disease worldwide after heart disease [1]. A cancer cell evolves from normal due to genetic mutations, which abnormally alter the cell proliferation rate. In particular, glioma is a rapidly evolving type of brain cancer, well known for its aggressive and diffusive behavior [2]. This diffusive invasion has lead several research efforts to explore the glioma’s progression with the aid of mathematical diffusion equations [3-5], aiming to predict its spatial and temporal evolution. The high diffusion rate of glioma cells from the core tumor into the surrounding brain tissue often leads to treatment failure and tumor recurrence, even after the surgical resection. Gliomas vary from low- to high-grade, namely glioblastomas, which constitute the most malignant form of brain cancer, having an extremely poor prognosis.

Several diverse factors influence glioma progression. Tumor cell migration in brain can be stimulated by the surrounding extracellular matrix (ECM) that is a complex mixture of macromolecules, some of which like the collagens play a structural role and others, such as laminin, fibronectin and vitronectin are important for cell adhesion, spreading and motility. ECM is remodeled by glioma cells that are able to degrade the surrounding tissue, through the secretion of matrix-degradative enzymes (MDEs), such as the plasminogen activator and the family of matrix metalloproteinases. Essentially, all tumors go through an avascular stage of growth, where the nutrient (oxygen and glucose) supply is adequate. The nutrients are provided both by the intratumoral ECM and the surroundings of tumor via diffusion. In a future growth stage, the intratumoral ECM is completely degraded, while the surrounding extracellular material is not close enough to supply the entire tumor body with the necessary nutrients via diffusion. Thus the tumor area is divided into layers consisting of different cell populations, namely proliferating, hypoxic, hypoglycemic and necrotic regions of tumor cells. Fully developed tumors contain a necrotic core with absence of nutrients, surrounded by hypoxic and hypoglycemic rings with limited access to nutrients and an outward proliferating cell ring having full nutrient supply. Since the tumor dependents on nutrients diffusion, its avascular growth is limited. Further growth and proliferation leads to its vascular stage, where cancer cells enhance the existing vascular network through angiogenesis. Furthermore, the metastatic final tumor stage succeeds the vascular stage. Since cancer development comprises these distinct stages (avascular, vascular and metastatic), the majority of research efforts are concentrated on exploring each one of these stages separately. In particular, identifying the avascular tumor dynamics constitutes the first crucial step towards fully vascularized tumors investigation [6].

In parallel to identification of tumor characteristics, the prediction of tumor growth and diffusion can lead to useful insight into the disease dynamics, which may improve clinical outcomes. To this respect, several mathematical and computational models have appeared in the literature, which investigate the mechanisms that govern glioma’s progression and invasion, with the aim of predicting its future spatial and temporal evolution, with or without the effects of therapy [7]. The models may constitute valuable tools for assisting the clinical practice towards the optimal individualized treatment, while facilitating medical research analysis. Current modelling techniques focus on either simulating individual cell behavior, or modelling tumor of clinically significant size, exploring volume expansion [7,8]. Such models follow a discrete cell-based description that simulates cell growth, or a continuum framework that deals with the evolution of local cellular densities and is based on diffusion-reaction equations. Since both approaches reveal particular tumor aspects but also pose certain limitations, hybrid models have been developed to combine continuum and discrete variable descriptions, but they are still limited by model complexity and uncertainties in the mixing of models. Alternatively, the notion of multi-compartment continuum model appears to be effective in describing how subpopulations of various cell types proliferate and diffuse, while it remains computationally efficient [9].

Discrete models are able to incorporate cellular parameters and biological rules, as well as simulate the effects of chemotaxis, haptotaxis, cell to cell adhesion and address intracellular processes and intercellular communication issues. Cancer cells are usually represented by vectors of variables that determine their position and speed in space, their cell cycle phase, etc [10-13]. Moreover, these models are able to easily describe heterogeneous cell populations. However, they are computationally intensive and fail to be efficiently initialized with tumors of clinically significant size that already consist of over 1 billion cells. The discrete model of 1000 billion individual units is beyond the capability of most computers, even if simple interactions algorithms are exploited [14]. Thus, such models are limited to the exploration of small tumors, below the level of clinical detection.

Continuum models represent alternative approaches in exploring tumor progression dynamics. Rather than considering each cell as a discrete individual, they assume tumors as spatial distributions of cell densities. As a result, computational requirements are substantially reduced. If the population of tumor cells is obtained as a continuum variable, the system can be described at macroscopic level as a biological tissue sample. Continuum models are also capable of accounting for the dynamic changes of chemical ingredients (such as glucose, oxygen, MDEs, chemotherapeutic drugs, etc) in the brain tissue. Additionally, they can take into account brain tissue heterogeneity and anisotropy. However, they lack a capability to simulate specific cellular factors, such as the genetic fingerprint of tumor cells and discontinuous changes, e.g. epithelial changes important for the invasion of tumor cells in adjacent tissue. Additionally, they are less efficient than discrete models in describing cellular-scale flux factors such as chemotaxis, haptotaxis and cell to cell adhesion.

Continuum approaches are most usually implemented by means of reaction-diffusion equations [15], where the cell proliferation factor is alternatively expressed by the Gompertz law, the second order polynomial equation, or the simple linear function [5,16]. Recent developments in mathematical modelling of gliomas are extensively analyzed in [4]. First, a model of untreated glioma is described followed by the presentation of models including chemotherapy, or surgical resection. The modelling assumptions vary from relatively simple considerations dealing with homogeneous brain tissue, to those involving heterogeneous brain tissue with different diffusion rates of glioma cells in grey and white matter on a geometrically complex brain domain. In the approach of [16], a three-dimensional diffusion model has been developed to address brain heterogeneity and anisotropy issues. The study introduces a mathematical framework incorporating alternative proliferation rate schemes and presents a solution of the diffusion equation through different numerical approximation of finite differences. Brain heterogeneity and anisotropy issues are dealt in the glioma growth model of [17], which is applied on real data. The proportions of white and grey matter, as well as the diffusion tensors are extracted by normal brain atlases, avoiding DTI processing. The research effort in [18] supports the hypothesis that the change in pH of tumor microenvironment may provide an important mechanism for cancer invasion. Another reaction-diffusion model presented in [19], deals with the diffusive behavior of glioma growth. This homogeneous model includes the Neumann boundary conditions imposed by the skull on gliomas and especially on glioblastoma multiforme (GBM). In [20] growth and diffusion issues of U87 glioblastoma tumor spheroids are investigated. The model includes diffusion, proliferation and cell to cell adhesion parameters, which differ for each cell line. U87 glioma cells are also explored in [21], where a model that includes the role of chemotaxis, haptotaxis and adhesion is presented. According to this model cell migration is strongly depend on adhesion, haptotactic and chemotactic parameters, apart from the random diffusion factor.

Expanding the potential of continuum mathematical models, some research efforts also engage the mechanical influence of tumor cells on the invaded tissue[5,22-26]. Many continuum approaches exploit the exponential Gompertz law [27] in order to model the proliferation of cancer cells. According to this law, the growth rates of cell populations are high at early stages of growth, while they are slower at later stages. The Gompertz tumor growth models take into account the fact that as the tumor size increases, the growth rate is reduced because of the reduction of available space and the limited supply of nutrients. The total cell population follows the Gompertz growth model in [28], where a two-compartment model of cancer cells population dynamics is proposed. The model consists of a set of ordinary differential equations, which simulate proliferating and quiescent cell populations including the transition rates between them. Moreover, Gompertz law and Gompertzian parameters available in the literature are exploited in the models of [29] and [30].

Recently, hybrid models have been developed to overcome particular limitations of distinct approaches. These models exploit the continuum method in order to simulate the tumor microenvironment, while cell to cell interactions are implemented using the discrete approach [14,31-37]. Some of these models are multiscale, incorporating the molecular and cellular level [6,38-40]. However, hybrid models are still constrained by the discrete-model limitations in which they do not consider tissue-level associations and they still have a significant computational cost.

Multi-compartment continuum models form alternative continuum approaches that are expected to overcome some of the conventional modelling limitations and are able to include multiphase heterogeneous populations and heterogeneous extracellular matrix [41]. According to this model philosophy, cells are grouped based on phenotype depending on their access to the necessary nutrients, or the level of differentiation [42]. Such approaches are computationally efficient and effective in describing how subpopulations of various types of cells proliferate and diffuse. Invasion, proliferation, changes in phenotype and necrosis are readily expressed as additions or subtractions from the cell densities within each compartment. In [3] a continuum multi-compartment model is proposed investigating how tumor cells interact with their tissue microenvironment. The dispersal and interactive population changes of normoxic and hypoxic glioma cells, vascular endothelial cells, diffusible angiogenic factors and necrotic cells are implemented using diffusion equations. Furthermore, the study demonstrates how different proliferation and diffusion rates of glioma cells result to increasing degrees of mitoses, hypoxia-induced neoangiogenesis and necrosis, features that characterize the different glioma grades.

In this paper, a new continuum three-dimensional spherical and multi-compartmental mathematical model of avascular glioma growth in an isotropic and homogeneous medium is proposed. This model expands the multi-compartmental approach of [3] by integrating a new cell compartment that represents the hypoglycemic cell population. The initial model is further extended through the exploitation of the microenvironmental variables of the hybrid approach in [32], where apart from oxygen, we introduce a second vital nutrient, (i.e. glucose). The proposed model consists of heterogeneous tumor cell populations incorporating the interactions between four different glioma cell phenotypes into distinct cellular compartments, namely proliferative, hypoxic, hypoglycemic and necrotic, as well as their tissue microenvironment. Moreover, the model includes the effect of the host tissue, i.e. the extracellular matrix (ECM), the matrix-degradative enzymes (MDEs) and nutrients (oxygen and glucose) concentration on tumor cell proliferation and invasion, through different microenvironmental compartments. The proposed approach employs continuum variables (cell densities, concentrations of chemical ingredients) governed by diffusion principles. It incorporates the effects of concentration for both important nutrients in the tumor microenvironment, namely oxygen and glucose. Notice that the majority of existing approaches deal with a single nutrient, which most often stands for oxygen [14,21,24,26,32,35,37,40],[43] and causes the development of hypoxic cell regions. Furthermore, existing approaches employing both vital nutrients (e.g. [1,6,34,39,44]) consider only the combined effect of their availability, which accounts for the generation of only quiescent (non-proliferating) and necrotic cells. The novelty of our proposed model lies in the fact that apart from the combined effect, it takes into consideration the separate and independent impact of each nutrient in tumor growth, which leads to the formation of a new cell population (apart from hypoxic, or quiescent and necrotic), namely hypoglycemic cells, with a different metabolic profile. Moreover, this model includes innovative variation of particular factors, such as the local cell proliferation rate, the variable conversion rates of cells from one category to another and the nutrient-dependent thresholds of conversion. The model is validated through a comparison with specific experimental results of glioma models available in the literature and additional evaluation by clinical experts. The results demonstrate the model’s efficiency, providing an essential tool for the patient-specific simulation of different tumor evolution scenarios and reliable prognosis of glioma spatio-temporal progression.

The paper is organized as follows. In Section “Mathematical modelling”, the different cell and chemical compartments are described in detail as building blocks of the model, coupled with the appropriate differential equations. Section “Results and model validation” presents and discusses experimental results of tumor growth for different combinations of diffusion and proliferation rates and for various evolution times. Moreover, model validation is performed through comparisons with experimental results of glioma models available in the literature, as demonstrated in the same section. Finally, significant conclusions are drawn in Section “Discussion and conclusions”, where potential future work aiming to model improvement and completion is also discussed.

## Mathematical modelling

Current glioma modelling techniques, either apply discrete cell-based tumor growth simulations focusing in individual cell behavior, or follow continuum approaches dealing with the evolution of tumor cell densities. The discrete models are computationally feasible only if the initial number of tumor cells is relatively small, which is not possible for a tumor of clinically significant size that is detectable in current imaging techniques (MRIs, CTs, etc). Taking this into consideration, the rationale behind choosing to develop a continuum glioma evolution model in this paper relates to the need of dealing with virtual tumors of clinically visible size and density, consisting of large numbers of cancer cells. This model is initialized by a virtual spherical glioma tumor and simulates multiple cellular-microenvironmental interactions aiming to predict its three-dimensional spatio-temporal evolution.

The evolution of glioma can be described by the following reaction diffusion equation [4,5].

(1) $$\frac{\mathrm{\partial C}}{\mathrm{\partial t}}=\nabla \cdot J(C,t)+S(C,t)-T(C,t)$$

where *J* represents the flux/motility of tumor cells, *S* stands for their net proliferation and *T* is the factor concerning the death of cancer cells due to microenvironmental conditions (e.g. absence of nutrients) and the beneficial effect of the applied treatment.

The motility of the tumor cells can be expressed as the result of four different fluxes *J*=*J*_*Random*_+*J*_*Haptotaxis*_+*J*_*Chemotaxis*_+*J*_*Adhesion*_, namely the random cell diffusion, as well as the diffusion due to haptotaxis, chemotaxis and cell to cell adhesion [20,21]. *J*_*Random*_=*D*_*R*_∇*C* concerns the random cell diffusion, which is constant in a homogeneous medium and *J*_*Haptotaxis*_=*D*_*Hapt*_·*C*∇*f* stands for the haptotaxis that is the directed migratory response of cells to gradients of non-diffusible chemicals, such as the extracellular matrix. Moreover, *J*_*Chemotaxis*_=*D*_*Chem*_·*C*∇*Gl* is the tendency of cancer cells to move in the direction of the nutrients (glucose) gradient and *J*_*Adhesion*_=*C*·*K*(*C*) corresponds to the movement due to cell to cell adhesion, according to which cells adhere to each other when they are close, while push apart when they are too compressed. In these equations *D*_*R*_, *D*_*Hapt*_, *D*_*Chem*_, are the random diffusion, the haptotactic and the chemotactic coefficient respectively, *C*, *f*, *Gl* the corresponding cancer cells, extracellular matrix and glucose concentrations and *K* represents the adhesive force of tumor cells.

The net proliferation rate of tumor cells *S* can be described by either a simple linear function *S*(*C*,*t*)=*ρ*·*C*, the second order polynomial equation $S(C,t)=\rho \cdot C\cdot (1-\frac{C}{C_{m}})$, or the Gompertz law $S(C,t)=\rho \cdot C\cdot \text{ln}(\frac{C_{m}}{C})$[5,16], where *C* is the concentration of cancer cells, *ρ* corresponds to the proliferation rate constant and *C*_*m*_ stands for the tissue cell maximum carrying capacity.

Cancer cells are capable of degrading the surrounding tissue, i.e. the extracellular matrix (ECM), which constitutes a crucial part of the invasive process. This degradation is achieved by means of the matrix-degradation enzymes (MDEs) that are produced by the cells and governed by the diffusion-reaction equation [32].

(2) $$\frac{\mathrm{\partial m}}{\mathrm{\partial t}}=D_{m}\nabla^{2}m+\mu \cdot C-\lambda \cdot m$$

where *m* represents the concentration of MDEs, *D*_*m*_ is the MDEs diffusion coefficient, *C* stands for the concentration of cancer cells, *μ* is the MDEs production rate and *λ* stands for the MDEs natural decay rate. The concentration change of ECM is simulated by the following equation [32].

(3) $$\frac{\mathrm{\partial e}}{\mathrm{\partial t}}=-\delta \cdot m\cdot e$$

where *e* represents the concentration of ECM and *δ* is the ECM degradation rate.

The tumor cells in order to survive, proliferate and diffuse need nutrients including oxygen and glucose. Many of them follow the anaerobic metabolism (glycolysis) instead of the normal aerobic metabolism that utilizes oxygen, even under of adequate oxygen presence [6,45]. The cells that follow the glycolytic pathway do not use oxygen; however they need a much larger quantity of glucose, in order to produce energy, in comparison to those that have an aerobic metabolism [34]. Hence, the tumor requires both oxygen and glucose, which are provided by the blood vessels and diffuse to the tumor area through the intratumoral and surrounding ECM. The nutrient production, diffusion and consumption are expressed by the diffusion-reaction equation [32].

(4) $$\frac{\mathrm{\partial n}}{\mathrm{\partial t}}=D_{n}\nabla^{2}n+\beta \cdot e-\gamma \cdot C-\alpha \cdot n$$

where *n* represents the concentration of the vital nutrient, *D*_*n*_ is the nutrient diffusion coefficient, *β* is the nutrient production rate through the ECM, *γ* stands for the nutrient consumption rate by the cancer cells and *α* is its natural decay rate.

Based on these equations, the proposed model expands the idea of glioma cell diffusion and proliferation taking into consideration different cell categories according to their metabolic profile, as well as the critical effect of two vital nutrients, which are consumed by the tumor cells.

### Proposed model description: concepts and assumptions

The proposed continuum mathematical approach models the three-dimensional spherical spatio-temporal evolution of an avascular glioma in an isotropic and homogeneous medium. The model incorporates specific interactions between the heterogeneous populations of cancer cells and their microenvironment, which affect tumor growth and invasion. It takes into consideration the separate and independent effect of the concentration changes for both of the important vital nutrients in the tumor microenvironment, namely oxygen and glucose, leading to the formation of distinct cell populations with different metabolic profile and invasion properties. This innovative feature of the proposed cancer model can deal with different cell-profile changes for each nutrient, thus, allowing the formation of different areas in the cancer neighborhood. In other related approaches, when the concentration of one of the two nutrients is sufficiently reduced (no matter which) only one new cell population (e.g. quiescent cells) appears before leading to necrosis. The proposed model consists of continuous variables, described by diffusion equations. The invasion of tumor cells, which is governed by the diffusion principles is based on the following equation:

(5) $$\frac{\mathrm{\partial C}(r,\theta ,\phi ,t)}{\mathrm{\partial t}}=D\nabla^{2}C(r,\theta ,\phi ,t)+f(C(r,\theta ,\phi ,t))$$

where *C* is the concentration of cancer cells at tumor point (*r*,*θ*,*ϕ*) (spherical coordinates) at time *t*, *D* represents the coefficient of random cell diffusion and *f* stands for the cell net proliferation rate. The proliferation rate in this model is based on the second order polynomial equation, since it takes in account the ratio of the local cell density and the maximum carrying capacity of the tissue, in order to limit the cell proliferation. Thus, when the tissue reaches its maximum carrying capacity, the tumor cells in this region, stop proliferating and remain quiescent, which is reasonable due to the lack of space. Furthermore, our model allows for variable local-cell proliferation rate due to the nutrients availability.

(6) $$f(C(r,\theta ,\phi ,t))=\rho \cdot C(r,\theta ,\phi ,t)\cdot (1-\frac{C(r,\theta ,\phi ,t)}{C_{m}})$$

In order to decrease the complexity of the model, we express the invasion of tumor cells only due to random diffusion, i.e. haptotaxis, chemotaxis and cell to cell adhesion have been neglected. The latter mechanism, according to which cells exert adhesive forces to each other, is observed only on the cellular scale. Since our model deals with the macroscopic consideration of cell densities, it is reasonable to ignore these forces. Moreover chemotaxis, namely the tendency of cancer cells to move in the direction of the nutrients, has been neglected in the current model. This consideration does not significantly degrade the approximation of real tumor cell-motility, since the tendency of the cells to move in the direction of nutrients supply is implicitly taken into account in our model. More specifically, due to random diffusion, the tumor cells tend to move from the areas of large cell concentrations towards less crowded regions, which however hold higher nutrient concentrations because of their lower consumption rate in comparison to the regions of large cell populations; hence, tumor cells are indirectly forced move towards nutrients. Similarly, the effects of haptotaxis, which is the directed migratory response of cells towards non-diffusible chemicals, e.g. ECM, are implicitly considered; through random diffusion, cells tend to move towards regions of lower densities, which happen to hold higher concentrations of ECM.

The proposed continuum model of spherical shape is multi-compartmental. The evolution of tumor mass is modeled by means of coupled diffusion equations, which simulate the different cell densities. The compartmental approach is exploited to track glioma cell subpopulations based on viability and phenotype. Thus, this model includes a proliferative region, along with a hypoxic, hypoglycemic and a necrotic one. The cellular size of all phenotypes is assumed to be the same and the cell diffusion coefficients are considered constant, since the tumor is assumed to evolve inside an isotropic and homogeneous medium. Additionally, coupled continuum components are developed in order to account for the changes in the local environment that affect cells within each compartment. These components concern the diffusion and consumption of vital nutrients, concentration and degradation of the host tissue, i.e. ECM, as well as production and diffusion of MDEs. The diffusion coefficients of nutrients and MDEs are assumed constant within all tumor heterogeneous regions and outside the tumor as well. The incorporated nutrients that determine tumor cell viability and affect proliferation are the oxygen and glucose.

Oxygen and glucose are transported to the tumor region from blood vessels and diffuse through the ECM located within the tumor and its surroundings, which is destroyed by MDEs produced by the tumor cells as the tumor grows. Initially, the size and cell density of the tumor are small, thus all cells are sufficiently supplied with oxygen and glucose through the intratumoral and surrounding extracellular material [1,6,21,26,31,36]. At this time all cancer cells are normoxic and proliferative, since the diffusion of nutrients is adequate for their survival and proliferation and most of them follow the aerobic metabolism. Furthermore, these cells compete for space with other neighboring cells; if the local tissue reaches its maximum carrying capacity, they stop proliferating and remain quiescent, while additionally they reduce their metabolism [32].

As time proceeds, the tumor radius increases and the concentrations of oxygen and glucose decrease in the central part of the tumor, closer to its core. At a certain time, each nutrient reaches a critical concentration value, below which it is not sufficient to meet the needs of all cells. Nutrients do not simultaneously fall below their critical concentrations, but are depleted independently depending on their consumption rates. Once the first nutrient, say oxygen, attains its critical concentration, the local tumor cells become hypoxic at a specific rate; a hypoxic region appears in the tumor’s center. Similarly, when the concentration of the other nutrient, i.e. glucose, falls below a critical level insufficient to meet energy needs of all cells, local cells turn to hypoglycemic and a hypoglycemic region appears in the central part of the tumor. The hypoxic and hypoglycemic cell populations neither proliferate nor die, while their metabolism changes. Specifically, the hypoxic cells perform increased glycolysis [45], which requires much less oxygen; the amount of oxygen consumed in hypoxic regions is not precisely known and could actually reach five times less the oxygen consumption in proliferative cells [32,46]. However, since anaerobic metabolism is far less efficient in producing energy compared to the normal aerobic one, hypoxic cells eventually consume over 10 times more glucose for maintaining the same energy turn-over [34]. As far as hypoglycemic cells are concerned, they reduce their metabolism, i.e. consumption of nutrients [6,32,35] and remain quiescent (they stop proliferating). Moreover, hypoxic cells are more migratory than normoxic proliferative cells [42], thus their diffusion coefficient is higher while they secrete larger amounts of degrading enzymes. When hypoxic cells migrate to an area with adequate oxygen, they convert back to normoxic at a specific constant rate; exactly the same applies to the case of the hypoglycemic cells, with respect to glucose [3]. The cells that remain in a state of oxygen and glucose adequacy in a hypoxic or a hypoglycemic area continue proliferating; however, as time proceeds these cell populations decrease, since they increasingly convert to hypoxic or hypoglycemic respectively. Eventually, in the hypoxic and/or hypoglycemic areas the total proliferation rate is reduced to the appropriate levels. As the tumor evolves, both hypoxic and hypoglycemic populations grow in the central part of the tumor at the expense of proliferative cells, to finally form a hypoxic and/or a hypoglycemic core within the tumor.

When the vast majority of cells have turned to hypoxic or hypoglycemic in the central part of the tumor, one of the nutrients (oxygen or glucose) tends to vanish and tumor cells start dying, i.e. become necrotic. Thus a necrotic region appears close to the tumor core, where nutrients can no longer diffuse due to the long distance from the tumor surroundings. Additionally, necrotic cells appear in a tumor area where both nutrients have achieved their critical concentrations, regardless of hypoxic and/or hypoglycemic cell proportion. Actually, any of the nutrients can be the first to extinct at the tumor center depending on the initial concentrations, their diffusion coefficients and consumption rates. The necrotic cells do not have a metabolism, do not proliferate and do not move, since they are not alive. Moreover, necrosis can also result from the viable proliferative cells due to their contact with the necrotic ones, at a particular rate [3]. In addition to necrosis, the natural cell death process is also present in the form of, apoptosis [1]. However, apoptosis has been neglected in this model as to keep the complexity to a manageable level, since the apoptosis rate is low enough in comparison to the necrosis rate and does not significantly affect the cell proliferation and invasion processes.

#### Model equations for tumor-cell densities

**Figure 1:**
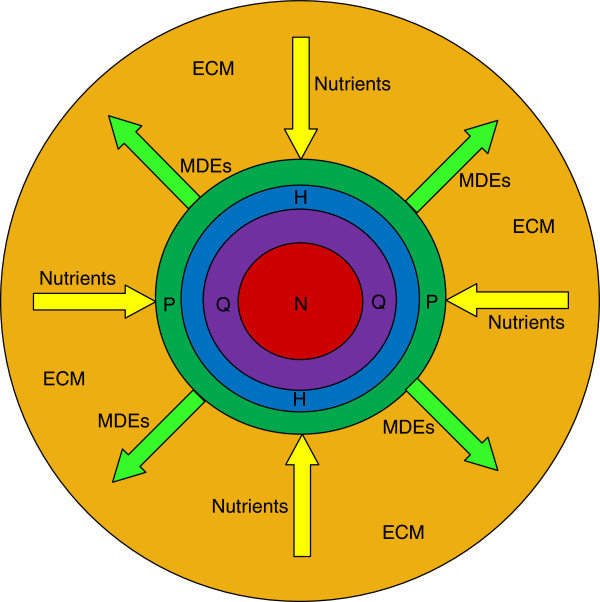
**Tumor regions in avascular evolution.** Tumor regions in avascular evolution, where P is the Proliferative, H is the Hypoxic, Q is the Hypoglycemic and N is the Necrotic zone.

Based on the above considerations of tumor progression, our model eventually considers tumor volume formed in four areas as in Figure 1, composed of the necrotic core, the hypoxic along with hypoglycemic rings around it, and the outer proliferating ring. Each of these areas consists mostly of cells belonging to the corresponding category. As long as the tumor radius increases, the hypoxic, hypoglycemic and necrotic radii increase as well. The tumor reaches a steady state when the hypoxic, hypoglycemic and necrotic regions occupy most of the entire tumor body.

In the model of Figure 1, four distinct glioma cellular compartments are developed. Each compartment constitutes a continuum equation, coupled with the remaining cellular compartments via common factors between them. The invasion of proliferative cancer cells follows the diffusion-reaction equation. The cell proliferation rate is expressed by the second order polynomial function, which takes into consideration the tissue maximum carrying capacity and diminishes the rate inversely according to the local cell density raise. As mentioned before, in a region of maximum cell density, the tumor cells apart from the fact that they do not proliferate, they reduce their metabolism (i.e. nutrient consumption) as well. Although these quiescent cells have temporarily stopped proliferating, they are included in the population of proliferative cells, since they still stay on a normoxic and normoglycemic state just as proliferative cells. The cell density equations are based on [3] and have been expanded to include the new cell compartment that represents the hypoglycemic population, as well as the effect of oxygen and glucose concentration in cell type (proliferative, hypoxic, hypoglycemic, necrotic) and proliferation rate. The equations governing the growth and invasion of proliferative cells are presented below.

(7) $$\begin{array}{c} \frac{\mathrm{\partial C}(r,\theta ,\phi ,t)}{\mathrm{\partial t}}=\nabla (D_{c}\cdot (1-T)\nabla C(r,\theta ,\phi ,t))+f(C(r,\theta ,\phi ,t))\\ \;+H(r,\theta ,\phi ,t)\cdot g_{h}\cdot (1-n_{h})+Q(r,\theta ,\phi ,t)\cdot g_{q}\cdot (1-gl_{q})\\ \;-C(r,\theta ,\phi ,t)\cdot b_{h}\cdot n_{h}-C(r,\theta ,\phi ,t)\cdot b_{q}\cdot gl_{q}\\ \;-C(r,\theta ,\phi ,t)\cdot N(r,\theta ,\phi ,t)\cdot a_{n} \end{array}$$

This equation involves several terms describing the various cellular processes discussed above, where *C*, *H*, *Q*, *N* denote the concentrations of proliferative (normoxic/normoglycemic), hypoxic, hypoglycemic and necrotic cancer cells respectively at tumor point (*r*,*θ*,*ϕ*), at time *t*, *D*_*c*_ represents the coefficient of random proliferative cell diffusion and *T* stands for the fraction of the local cell density to the maximum carrying capacity, given by:

(8) $$T=\frac{C(r,\theta ,\phi ,t)+H(r,\theta ,\phi ,t)+Q(r,\theta ,\phi ,t)+N(r,\theta ,\phi ,t)}{C_{m}}$$

Furthermore, *b*_*h*_, *b*_*q*_ are the conversion rates of proliferative cells to hypoxic and hypoglycemic respectively, which unlike [3] are variable proportional to the oxygen or glucose relative concentrations. Additionally, *a*_*n*_ stands for the constant of conversion rate of proliferative cells (as well as hypoxic and hypoglycemic) to necrotic due to the contact with the necrotic region and *g*_*h*_, *g*_*q*_, are the constant conversion rates of hypoxic and hypoglycemic cells to proliferative, respectively. Moreover, *n*_*h*_, *gl*_*q*_ represent the thresholds for the proliferative cells to turn to hypoxic or hypoglycemic and in this model are related to the critical nutrients concentrations. The function *f* in Equation 7 stands for the local proliferation rate of tumor cells and corresponds to an innovative net proliferation rate variation, since in our model depends on the local oxygen and glucose availability. It can be modeled as:

(9) $$f(C(r,\theta ,\phi ,t))=O_{\text{con}}\cdot Gl_{\text{con}}\cdot \rho \cdot C(r,\theta ,\phi ,t)\cdot (1-T)$$

where *ρ* corresponds to the proliferation rate constant and *O*_*con*_, *Gl*_*con*_ are coefficients that reduce the proliferation rate in relation to the local oxygen (*n*) and glucose (*gl*) concentration respectively and are given as in the following equations. The concentrations *n* and *gl* have been calculated including the current consumption by the cells at each time point, while *n*_*tmp*_ and *gl*_*tmp*_ are the corresponding concentrations before nutrient consumption. Additionally, *n*_0_ and *gl*_0_ represent the maximum concentrations of the nutrients which initially exist in the tumor surroundings. In particular,

(10) $$\;O_{\text{con}}=1-(b_{h}+a_{n})\cdot n_{h},\;\;n_{h}=\left\{\;\begin{array}{cc} 0, & n(r,\theta ,\phi ,t)>0\\ 1, & n(r,\theta ,\phi ,t)\leq 0 \end{array}\right)\;\;\text{and}\;\;b_{h}=(1-\frac{n_{\text{tmp}}(r,\theta ,\phi ,t)}{n_{0}})/20$$

(11) $$\;Gl_{\text{con}}=\;1-(b_{q}+a_{n})\cdot gl_{q},\;\;gl_{q}=\;\left\{\;\begin{array}{cc} 0, & \text{gl}(r,\theta ,\phi ,t)>0\\ 1, & \text{gl}(r,\theta ,\phi ,t)\leq 0 \end{array}\;\;\text{and}\;\;b_{q}=(1-\frac{gl_{\text{tmp}}(r,\theta ,\phi ,t)}{gl_{0}})/20\right)$$

The invasion of hypoxic cells is governed by diffusion, following the equation below:

(12) $$\begin{array}{c} \frac{\mathrm{\partial H}(r,\theta ,\phi ,t)}{\mathrm{\partial t}}=\nabla (D_{h}\cdot (1-T)\nabla H(r,\theta ,\phi ,t))\\ \;+C(r,\theta ,\phi ,t)\cdot b_{h}\cdot n_{h}-H(r,\theta ,\phi ,t)\cdot g_{h}\cdot (1-n_{h})\\ \;-H(r,\theta ,\phi ,t)\cdot a_{h}\cdot n_{\text{hn}}-H(r,\theta ,\phi ,t)\cdot a_{\text{glh}}\cdot gl_{\text{hn}}\\ \;-H(r,\theta ,\phi ,t)\cdot N(r,\theta ,\phi ,t)\cdot a_{n} \end{array}$$

where *D*_*h*_ represents the coefficient of random hypoxic cell diffusion, *a*_*h*_ is the variable (proportional to *b*_*h*_) conversion rate of hypoxic cells to necrotic due to the lack of oxygen, while *a*_*glh*_, is the conversion rate of hypoxic cells to necrotic, due to the absence of glucose, *n*_*hn*_, *gl*_*hn*_ represent the thresholds for the hypoxic cells to turn to necrotic due to the absence of oxygen, or glucose respectively, modeled as:

$$n_{\text{hn}}=\left\{\begin{array}{cc} 0, & \frac{H(r,\theta ,\phi ,t)}{C(r,\theta ,\phi ,t)+H(r,\theta ,\phi ,t)+Q(r,\theta ,\phi ,t)}\leq 0.9\\ 1, & \frac{H(r,\theta ,\phi ,t)}{C(r,\theta ,\phi ,t)+H(r,\theta ,\phi ,t)+Q(r,\theta ,\phi ,t)}>0.9 \end{array}\right)\;\text{and}\;gl_{\text{hn}}=\left\{\begin{array}{cc} 0, & n_{h}=1,\;gl_{q}=0\\ 1, & n_{h}=1,\;gl_{q}=1 \end{array}\right)$$

Similarly the diffusion of hypoglycemic cells is expressed by the following equation:

(13) $$\begin{array}{c} \frac{\mathrm{\partial Q}(r,\theta ,\phi ,t)}{\mathrm{\partial t}}=\nabla (D_{q}\cdot (1-T)\nabla Q(r,\theta ,\phi ,t))\\ \;+C(r,\theta ,\phi ,t)\cdot b_{q}\cdot gl_{q}-Q(r,\theta ,\phi ,t)\cdot g_{q}\cdot (1-gl_{q})\\ \;-Q(r,\theta ,\phi ,t)\cdot a_{q}\cdot n_{\text{qn}}-Q(r,\theta ,\phi ,t)\cdot a_{\text{glq}}\cdot gl_{\text{qn}}\\ \;-Q(r,\theta ,\phi ,t)\cdot N(r,\theta ,\phi ,t)\cdot a_{n} \end{array}$$

where *D*_*q*_ represents the coefficient of random hypoglycemic cell diffusion, *a*_*q*_ is the conversion rate of hypoglycemic cells to necrotic due to the lack of oxygen, while *a*_*glq*_ is the variable (proportional to *b*_*q*_) conversion rate of hypoglycemic cells to necrotic, due to the absence of glucose. Furthermore, *n*_*qn*_, *gl*_*qn*_ represent the thresholds for the hypoglycemic cells to turn to necrotic due to the absence of oxygen or glucose respectively, described in the form:

$$\;gl_{\text{qn}}=\left\{\begin{array}{cc} 0, & \frac{Q(r,\theta ,\phi ,t)}{C(r,\theta ,\phi ,t)+H(r,\theta ,\phi ,t)+Q(r,\theta ,\phi ,t)}\leq 0.75\\ 1, & \frac{Q(r,\theta ,\phi ,t)}{C(r,\theta ,\phi ,t)+H(r,\theta ,\phi ,t)+Q(r,\theta ,\phi ,t)}>0.75 \end{array}\right)\;\text{and}\;n_{\text{qn}}=\left\{\begin{array}{cc} 0, & gl_{q}=1,\;n_{h}=0\\ 1, & gl_{q}=1,\;n_{h}=1 \end{array}\right)$$

Finally, necrotic cells do not diffuse and thus their concentration changes follow the rather simple equation:

(14) $$\begin{array}{c} \frac{\mathrm{\partial N}(r,\theta ,\phi ,t)}{\mathrm{\partial t}}=C(r,\theta ,\phi ,t)\cdot N(r,\theta ,\phi ,t)\cdot a_{n}\\ \;+H(r,\theta ,\phi ,t)\cdot a_{h}\cdot n_{\text{hn}}+H(r,\theta ,\phi ,t)\cdot a_{\text{glh}}\cdot gl_{\text{hn}}\\ \;+H(r,\theta ,\phi ,t)\cdot N(r,\theta ,\phi ,t)\cdot a_{n}+Q(r,\theta ,\phi ,t)\cdot a_{q}\cdot n_{\text{qn}}\\ \;+Q(r,\theta ,\phi ,t)\cdot a_{\text{glq}}\cdot gl_{\text{qn}}+Q(r,\theta ,\phi ,t)\cdot N(r,\theta ,\phi ,t)\cdot a_{n} \end{array}$$

The factors participating in Equation 14 have all been mentioned above.

#### Model equations for nutrient concentrations

The concentration-changes of oxygen and glucose are similarly governed by diffusion equations, which includes their production through the ECM, their natural decay and their consumption by the tumor cells. The oxygen concentration equation, as well as the new glucose concentration compartment is based on [32]. Both of the nutrients equations have been expanded to involve the different consumption rates by each of the distinct cell types (proliferative, hypoxic and hypoglycemic). More specifically:

(15) $$\begin{array}{lll} \frac{\mathrm{\partial n}(r,\theta ,\phi ,t)}{\mathrm{\partial t}}=\; & D_{n}\nabla^{2}n(r,\theta ,\phi ,t)+\beta_{n}\cdot e(r,\theta ,\phi ,t)-\alpha_{n}\cdot n(r,\theta ,\phi ,t)\; & \;\\ & -\gamma_{\text{cn}}\cdot C(r,\theta ,\phi ,t)-\gamma_{\text{hn}}\cdot H(r,\theta ,\phi ,t)-\gamma_{\text{qn}}\cdot Q(r,\theta ,\phi ,t)\; \end{array}$$

(16) $$\begin{array}{lll} \frac{\mathrm{\partial gl}(r,\theta ,\phi ,t)}{\mathrm{\partial t}}=\; & D_{\text{gl}}\nabla^{2}\text{gl}(r,\theta ,\phi ,t)+\beta_{\text{gl}}\cdot e(r,\theta ,\phi ,t)\; & \;\\ & -\alpha_{\text{gl}}\cdot \text{gl}(r,\theta ,\phi ,t)-\gamma_{\text{cgl}}\cdot C(r,\theta ,\phi ,t)\; & \;\\ & -\gamma_{\text{hgl}}\cdot H(r,\theta ,\phi ,t)-\gamma_{\text{qgl}}\cdot Q(r,\theta ,\phi ,t)\; \end{array}$$

where *n*, *gl* represent the concentration of the oxygen, glucose at tumor point (*r*,*θ*,*ϕ*), at time *t*, *D*_*n*_, *D*_*gl*_ are the respective diffusion coefficients, *β*_*n*_, *β*_*gl*_ are the nutrients production rates through ECM, *α*_*n*_, *α*_*gl*_ are the nutrients natural decay rates, *γ*_*cn*_, *γ*_*cgl*_ stand for the nutrients consumption rates by the proliferative cancer cells, *γ*_*hn*_, *γ*_*hgl*_ are the nutrients consumption rates by the hypoxic cells and respectively *γ*_*qn*_, *γ*_*qgl*_ are the nutrients consumption rates by the hypoglycemic cells. In the tumor area where local cell-density has reached the tissue maximum carrying capacity, the normoxic/normoglycemic cells have been considered in this model to consume half of the nutrients’ amounts [32].

The concentration change of ECM because of its degradation by MDEs produced by the tumor cells, is demonstrated by the following equation (based on [32]):

(17) $$\frac{\mathrm{\partial e}(r,\theta ,\phi ,t)}{\mathrm{\partial t}}=-\delta \cdot m(r,\theta ,\phi ,t)\cdot e(r,\theta ,\phi ,t)$$

where *e* is concentration of ECM at tumor point (*r*,*θ*,*ϕ*), at time *t*, *m* represents the concentration of MDEs and *δ* is the ECM degradation rate. MDEs are subjected to the principle of diffusion, produced by proliferative, hypoxic and hypoglycemic cells and are characterized by a natural decay rate. The MDEs equation is based on [32] and has also been expanded to include the distinct tumor cell categories:

(18) $$\begin{array}{c} \frac{\mathrm{\partial m}(r,\theta ,\phi ,t)}{\mathrm{\partial t}}=D_{m}\nabla^{2}m(r,\theta ,\phi ,t)+\mu_{c}\cdot C(r,\theta ,\phi ,t)\\ \;+\mu_{h}\cdot H(r,\theta ,\phi ,t)+\mu_{q}\cdot Q(r,\theta ,\phi ,t)-\lambda \cdot m(r,\theta ,\phi ,t) \end{array}$$

where *D*_*m*_ is the diffusion coefficient of MDEs, *μ*_*c*_, *μ*_*h*_, *μ*_*q*_ are the production rates by the proliferative hypoxic and hypoglycemic cells respectively and *λ* represents the MDEs natural decay rate.

### Parameter estimation

One of the major limitations of mathematical models concerns the parameter initialization using literature-based reference values. These values are often difficult to obtain, since most of them are patient specific and vary significantly among clinical and experimental cases. However, the existing wide range of values for each parameter allows models to incorporate different abnormal cases and examine many alternative outcomes.

A continuum glioma model can be initialized based either on the actual tumor geometry derived from medical images, or on a virtual spherical tumor [1], which approximates a real one, in terms of its imaging detectable size. In imaging modalities such as CT and MRI, the tumor is detectable above a certain size and density [3,4]. According to the literature, the density threshold associated with the detectable boundary of a glioma in a medical image has been estimated to *C*_2*D*_=400*cells*/*mm*^2^, which leads to $C_{3D}={\left(\sqrt{C_{2D}}\right)}^{3}=8*10^{3}\text{cells}/mm^{3}$, while the smallest detectable size corresponds to a radius of *r*=1 *cm*. According to medical experts tumors of radius smaller than 1 *cm* (e.g., *r*=5 *mm*) can be diagnosed. Yet, taking into consideration that oxygenated cells are limited to a distance of less than 0.1−0.2 *mm* from blood vessels, most of the initially detectable tumors (*r*≥5 *mm*) should already contain at least a hypoxic, even a necrotic region. Thus, in order to address tumorogenesis at early stages, our model is initialized with a virtual tumor of a smaller radius (i.e. *r*=1 *mm*), under the assumption that the initial tumor consists only of one cell category, namely proliferating cells without a significant obliquity. Accordingly, the initial tumor density has been considered to be slightly higher than the corresponding minimum detectable threshold, i.e. *C*_0_=10^4^*cells*/*mm*^3^. This constitutes the initial condition for the current model, which involves two boundaries; the inner one (*r*=*r*_0_) corresponding to the tumor border and the outer boundary (*r*=*r*_1_) taken far enough from the tumor and defining the area of the medium to be invaded by the tumor. The initial condition for tumor cell density is described as:

(19) $$C(r,\theta ,\phi ,0)=\left\{\begin{array}{cc} C_{0}, & 0\leq r\leq r_{0}\\ 0, & r_{0}<r\leq r_{1} \end{array}\right)$$

The invasion rate of glioma varies among patients depending on the grade of the disease. A wide range of invasion rates has been observed in clinical experience from serial MRIs and can be found in the literature in the form of several diffusion coefficient values. Similarly, for another patient-specific parameter namely the constant of tumor cell proliferation rate, a wide range of values has been reported. Different combinations of the diffusion and proliferation rates determine the different glioma grades. For instance, concerning a four grade malignant glioma (glioblastoma), a primary one is characterized by low diffusion and high proliferation, while a glioblastoma progressing from lower grade is determined by higher diffusion but lower proliferation rate [3]. In this paper, various combinations of diffusion and proliferation rates have been considered, in order to explore different invasion and growth cases associated to various glioma grades. As far as the diffusion of hypoxic cells is concerned, since they are more migratory than normoxic cells [42], their invasion rate is higher; here their diffusion coefficient is taken one order of magnitude higher compared to the normoxic cells value. On the other hand, due to the lack of related data, the diffusion coefficient of hypoglycemic cells has been considered the same as the one of proliferative cells.

Initially, apart from outside the tumor, ECM is located within the tumor area as if it has not been completely degraded by MDEs. In our consideration, the initial intratumoral ECM concentration is considered one order of magnitude lower than the ECM maximum concentration at the tumor surroundings. As the tumor grows, intratumoral ECM is destroyed by MDEs, which are produced by the proliferative, hypoxic and hypoglycemic cells at specific rates. Since hypoxic cells are more invasive than normoxic cells, their MDEs secretion is considered larger and this is reflected in our model by accounting a twice higher MDEs production rate. Regarding hypoglycemic cells, they are associated to the same MDEs production rate as proliferative cells. The initial concentration of MDEs has been considered zero outside the tumor boundary and nonzero inside the tumor. More specifically, since the initial tumor density is lower than the tissue maximum carrying capacity, the initial intratumoral MDEs concentration is considered lower than the maximum reference value, equal to the ratio of the tumor density over the maximum carrying capacity.

The initial tumor is sufficiently provided with nutrients through the remaining intratumoral ECM, as well as from the surrounding ECM via diffusion. The maximum nutrients concentration exists in the area outside the tumor, which serves as the nutrient source. In the current model, nutrient concentrations are reduced towards the central part of the tumor [1] according to a normal distribution, depending on the distance from tumor periphery as demonstrated by the following equation:

(20) $$n_{0\text{tumor}},\;gl_{0\text{tumor}}=(n_{0},\;gl_{0})\cdot e^{-d_{r}}$$

where *n*_0_, *gl*_0_ represent the initial oxygen and glucose concentrations outside the tumor and *D*_*R*_ is the distance of the current point from tumor periphery. The diffusion, as well as production, consumption and natural decay-rate parameters of the different chemicals (i.e. nutrients, ECM and MDEs) are taken from related research studies, such as [1,3,21,32,33,35,46-49]. Particularly, regarding the metabolic rates of the various cell phenotypes, hypoglycemic cells are assumed to consume nutrients at half the rate of proliferating cells. On the contrary, since the vast majority of hypoxic cells are considered to follow the glycolytic pathway, they are assumed to consume 10 times more glucose, but 1/5 less oxygen [42], compared to normoxic cells, the majority of which (approximately 60%) are considered to follow the aerobic metabolism in this model. Some parameters were hard to obtain and they were estimated according to related known values. The parameter values that were used in the equations of our model are summarized in Table A1 contained in an additional file (please see Additional file 1).

Boundary conditions for all tumor cell densities are determined by the first set of the following equation pairs. The same boundary conditions apply for MDEs concentration. Finally, nutrients and ECM are determined by the second equation pair.

(21) $$\left\{\begin{array}{cc} \frac{\mathrm{\partial C}(r,\theta ,\phi ,t)}{\mathrm{\partial r}}=0, & r=0\\ C(r,\theta ,\phi ,t)=0, & r=r_{1} \end{array},\right)\;\left\{\begin{array}{cc} \frac{\mathrm{\partial n}(r,\theta ,\phi ,t)}{\mathrm{\partial r}}=0, & r=0\\ n(r,\theta ,\phi ,t)=n_{0}, & r=r_{1} \end{array}\right)$$

## Results and model validation

### Simulation results

**Figure 2:**
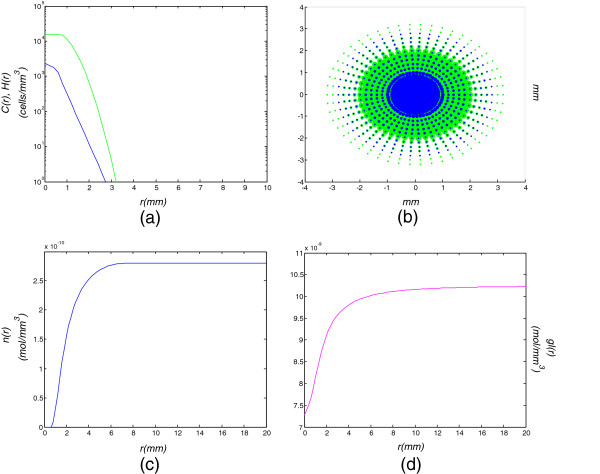
**Model simulation of a low diffusion-high proliferation tumor for 30 days (1 month).** Model simulation of a low diffusion-high proliferation tumor for 30 days (1 month): **(a)** cell density with respect to the tumor radius, **(b)** tumor cells dispersal in a 2D section, **(c)** oxygen and **(d)** glucose concentrations with respect to the tumor radius.

**Figure 3:**
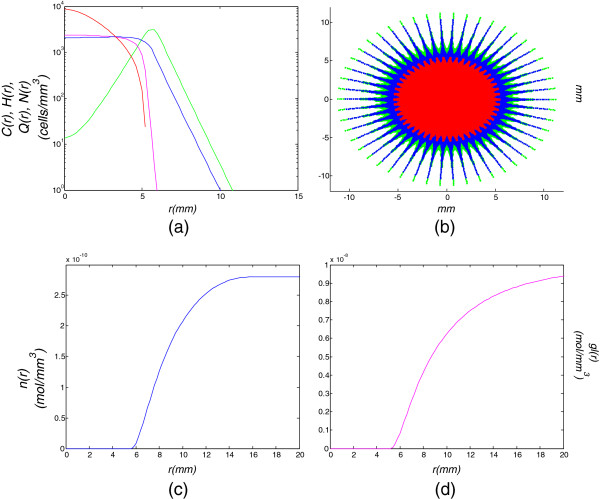
**Model simulation of a low diffusion-high proliferation tumor for 180 days (6 months).** Model simulation of a low diffusion-high proliferation tumor for 180 days (6 months): **(a)** cell density with respect to the tumor radius, **(b)** tumor cells dispersal in a 2D section, **(c)** oxygen and **(d)** glucose concentrations with respect to the tumor radius.

**Figure 4:**
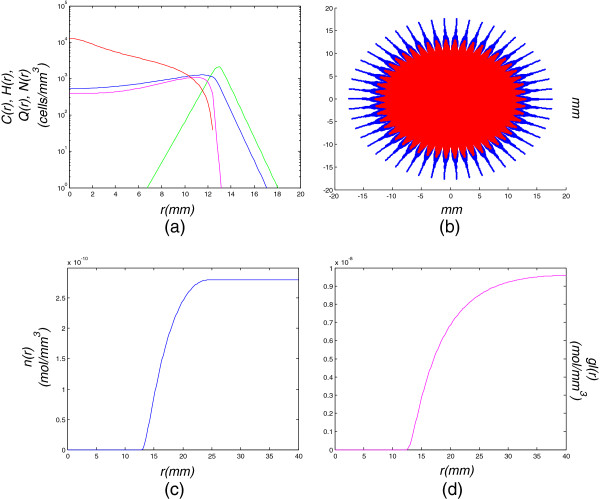
**Model simulation of a low diffusion-high proliferation tumor for 360 days (12 months).** Model simulation of a low diffusion-high proliferation tumor for 360 days (12 months): **(a)** cell density with respect to the tumor radius, **(b)** tumor cells dispersal in a 2D section, **(c)** oxygen and **(d)** glucose concentrations with respect to the tumor radius.

**Figure 5:**
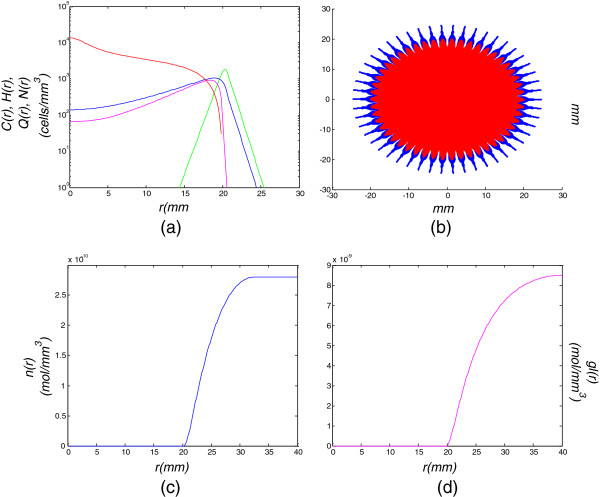
**Model simulation of a low diffusion-high proliferation tumor for 540 days (18 months).** Model simulation of a low diffusion-high proliferation tumor for 540 days (18 months): **(a)** cell density with respect to the tumor radius, **(b)** tumor cells dispersal in a 2D section, **(c)** oxygen and **(d)** glucose concentrations with respect to the tumor radius.

The compartmental equations of the proposed model along with its solutions have been numerically approximated by means of a finite differences method, namely Forward Euler. Several simulations have been performed using combinations of diffusion and proliferation rate constants, for the various evolution times of 1, 2, 3, 6, 9, 12, 15 and 18 months. More specifically, the results concern the combinations of [low diffusion, low proliferation], [high diffusion, low proliferation], [low diffusion, high proliferation], [high diffusion, high proliferation] and [medium diffusion, medium proliferation]. The model is initialized with an early-stage tumor of radius *r*_0_=1 *mm*, smaller than the minimum detectable size consisting only of proliferating cells, without any other assumptions on the initial radius of the various cell-population regions. Furthermore, the quiescent cells due to the lack of space have been included in the population of proliferative cells, since they still remain on normoxic and normoglycemic conditions. The simulation results of cell and nutrient densities with respect to the distance from the center of the tumor, for the combination of low diffusion (*D*=0.005 *mm*^2^/*day*) and high proliferation rate (*ρ*=0.04/*day*), corresponding to a primary high grade glioma, i.e. a glioblastoma from its first clinical detectability, are graphically illustrated in Figures 2, 3, 4 and 5 for the evolution times of 1, 6, 12 and 18 months. More results concerning the intermediate evolution times of 2, 3, 9 and 15 months are illustrated in an additional file (please see Additional file 2). This additional file also contains the graphical evolution of the low diffusion-high proliferation glioma evolution in the early stage of 1 day after its assumed detection, where all cancer cells remain proliferative since no hypoxia and/or hypoglycemia have appeared yet. Finally, all results concerning radius of regions for the various cell categories for the different times stages and the diffusion-proliferation pairs of interest are depicted in Table 1.

**Table 1:** Model simulation for 1, 2, 3, 6, 9, 12, 15 and 18 months

**Model simulation results**						
***t***	***D*(mm^2/day)**	***0.005***	***0.3***	***0.005***	***0.3***	***0.04***
**(*days*)**	***ρ*(/*day*)**	***0.0025***	***0.0025***	***0.04***	***0.04***	***0.015***
	*P*_*r*_	3.05	12	3.2	13.5	6.2
30	*H*_*r*_	0	0	2.7	0	0
	*Q*_*r*_	0	0	0	0	0
	*N*_*r*_	0	0	0	0	0
	*P*_*r*_	3.75	16	4.9	19	8.2
60	*H*_*r*_	0	0	5.1	0	0
	*Q*_*r*_	0	0	0.5	0	0
	*N*_*r*_	0	0	0.3	0	0
	*P*_*r*_	4.3	18	6.7	25	9.8
90	*H*_*r*_	0	0	6.7	0	0
	*Q*_*r*_	0	0	2.6	0	0
	*N*_*r*_	0	0	1.9	0	0
	*P*_*r*_	5.6	22	11	45	14
180	*H*_*r*_	0	0	10	42.5	0
	*Q*_*r*_	0	0	6.5	21.5	0
	*N*_*r*_	0	0	5.5	18	0
	*P*_*r*_	6.5	25	14.5	74.5	18.5
270	*H*_*r*_	0	0	13.5	69	14
	*Q*_*r*_	0	0	9.5	46	0
	*N*_*r*_	0	0	9	42.5	0
	*P*_*r*_	7.35	28	18	103	24
360	*H*_*r*_	0	0	17	95	21
	*Q*_*r*_	0	0	13.3	71	10
	*N*_*r*_	0	0	12.5	69	9
	*P*_*r*_	8.15	33	22	131	30
450	*H*_*r*_	0	0	21	123	26.5
	*Q*_*r*_	0	0	17	100	16
	*N*_*r*_	0	0	16.5	96	14
	*P*_*r*_	8.8	40	25.5	157	36
540	*H*_*r*_	0	0	24	149	32
	*Q*_*r*_	0	0	21	127	21.5
	*N*_*r*_	0	0	20	125	20

Radius (mm) of each cell population (Proliferative: *P*_*r*_, Hypoxic: *H*_*r*_, Hypoglycemic: *Q*_*r*_, Necrotic: *N*_*r*_) with respect to each diffusion-proliferation combination.

Figure 2 demonstrates the evolution of the primary glioblastoma 1 month after its assumed detection, when its radius has increased from *r*=1 *mm* to *r*=3.2 *mm*. It can be noticed that due to the high proliferation rate and the low invasion, cell density rapidly increases in the tumor center, which leads to the appearance of hypoxia (blue curve and region in Figures 2(a), 2(b)). The hypoxia is also verified in Figure 2(c) reflecting the oxygen inadequacy in the central part of the tumor. Concerning the other diffusion-proliferation combinations demonstrated in Table 1, the cells remain proliferative throughout the tumor, implying that there is still adequacy of oxygen and glucose. This proves that as D gets larger, the tumor is dominated by its diffuse extent that reduces cell density in its central part, thus it takes longer for the proliferative cells to fall into hypoxic and hypoglycemic conditions.

Simulation results of 6 months after the assumed tumor diagnosis (low diffusion, high proliferation) in Figure 3, demonstrate that the hypoxic cell density at the tumor center has increased. Additionally, a hypoglycemic cell population has appeared (magenta curve in Figure 3(a)) due to the glucose inadequacy, which is shown in Figure 3(d). Glucose concentration is further decreased since hypoxic cells consume much more glucose in order to survive. Apart from hypoxic and hypoglycemic cell populations, a necrotic core of approximately 3 mm, has been formed (red curve and region in Figures 3(a), 3(b)). This is followed by the hypoxic and hypoglycemic regions around it and the external proliferative region. Here the hypoglycemic zone is incorporated inside the hypoxic band. The hypoxic, hypoglycemic and necrotic populations have increased at the expense of proliferating cells (green curve in Figure 3(a)), which now constitute the minority for the tumor radius of *r*<5 *mm*. Moreover, at the same time hypoxic, hypoglycemic and necrotic regions have appeared for the high diffusion, high proliferation case (Table 1), where the tumor radius already exceeds the value of *r*=4 *cm*. This case could represent a progressive glioblastoma.

After 12 months (Figure 4), the hypoxic and hypoglycemic areas have grown while the separate zones have become more distinct, since the cells that compose each zone prevail on all other cells. It can be clearly observed that proliferating cells no longer exist in the central part of the tumor. Additionally, it is shown that the hypoglycemic region is still incorporated within the hypoxic one. Moreover, the glioma represented by medium diffusion, medium proliferation rates has progressed to grade IV, containing a necrotic central region, representing a secondary glioblastoma.

Finally, Figure 5 illustrates tumor growth after 18 months, where all the distinct regions have further increased in size and density from inside to outside. The formation of the extended necrotic core, surrounded by the hypoglycemic and hypoxic regions and the outward proliferating zone are clearly depicted.

**Figure 6:**
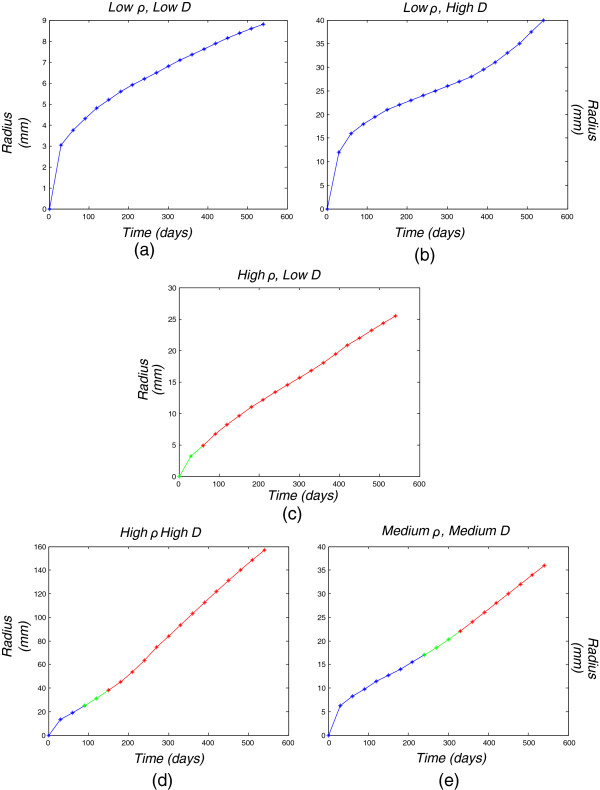
**Glioma grade as a function of tumor size and time.** Tumor grade as a function of tumor size and time for each of the five diffusion-proliferation (*D*−*ρ*) combinations: **(a)** low diffusion-low proliferation, **(b)** high diffusion-low proliferation, **(c)** low diffusion-high proliferation, **(d)** high diffusion-high proliferation, **(e)** medium diffusion-medium proliferation. Blue part: grade II radius, green part: grade III radius, red part: Grade IV radius.

Table 1 and Figure 6 demonstrate the relation between the diffusion coefficient and the tumor-growth rate. More specifically, it can be observed that in all cases the expansion rate is temporarily reduced after the first 3 months, while later it is increased proportionally to the diffusion-coefficient value concerning the medium and high diffusion cases, to finally reach a steady growth state. Regarding the low diffusion cases, the tumor growth rate is stabilized after its first reduction.

### Model validation

Model validation constitutes an essential part of mathematical modelling and it can be performed using either medical data stemming from clinical cases, or experimental model results available in the literature. The model in this paper is based on a virtual spherical tumor, so that its validation is more appropriate using experimental model results derived from similar existing research efforts. However, the same model can be initialized from a real tumor anatomy derived from MRI imaging. Nevertheless, the comparison with actual clinical data requires the use of nonhomogeneous media considerations, which is outside the scope of this paper. The results of the current study are compared to the corresponding outputs of the continuum model in [3] and the hybrid model in [1]. In both cases, high correlation was revealed between simulated and experimental results.

**Figure 7:**
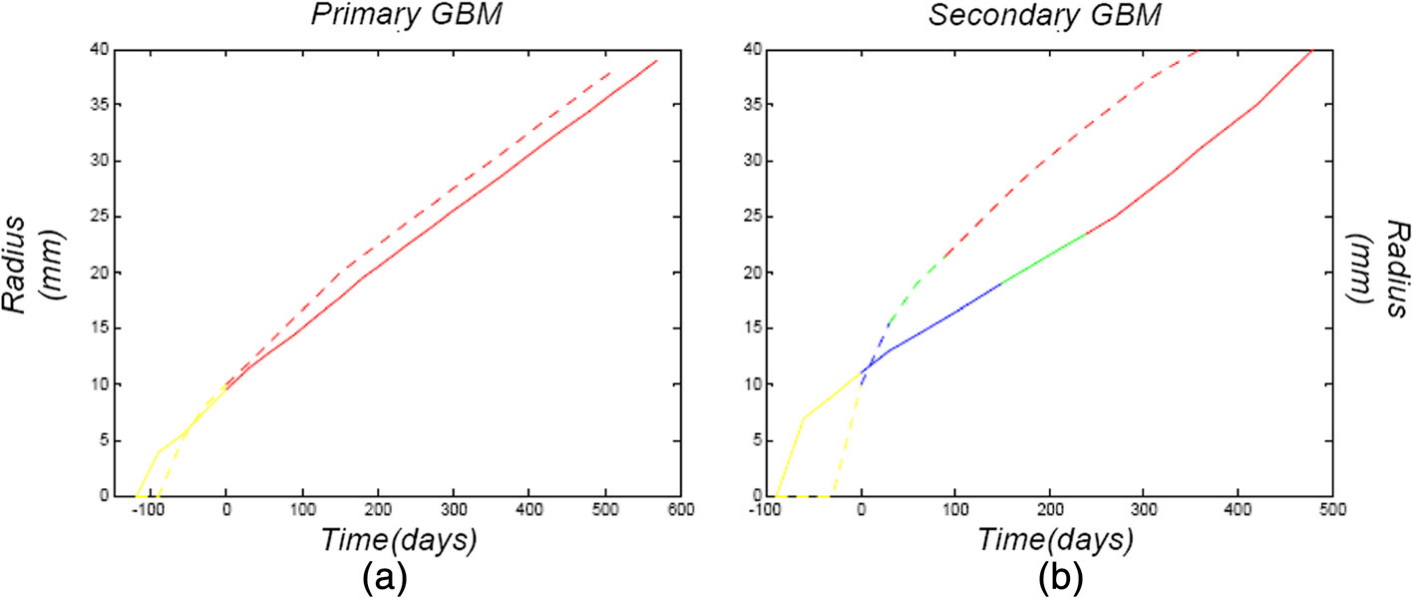
**Glioma grade as a function of tumor size and time in comparison to **[3]** results.** Tumor grade as a function of tumor size and time in comparison to results of [3] for two different diffusion-proliferation pairs. **(a)** corresponds to a primary glioblastoma produced by *D*=0.01 *mm*^2^/*day*, *ρ*=0.033/*day*, **(b)** illustrates a secondary (progressive from lower grade) glioblastoma produced by *D*=0.06 *mm*^2^/*day*, *ρ*=0.013/*day*. The dotted curves correspond to comparative results of [3]. Yellow part: minimum T2 detectable radius, blue part: grade II radius, green part: grade III radius, red part: grade IV radius.

First, the experimental results drawn from graphs presented in paper [3] are compared to simulations results of the proposed model. Grade association with size and time is depicted in Figure 7 for two different diffusion-proliferation combinations in comparison to respective results of [3]. Specifically, Figure 7(a) demonstrates a primary glioblastoma (grade IV from its first detectability) and corresponds to low diffusion and high proliferation rate, while Figure 7(b) simulates a secondary glioblastoma, (progressing to grade IV from lower grade) and stands for medium diffusion and medium proliferation rate. For both cases, the initial tumor has a density equal to *C*_0_=10^4^*cells*/*mm*^3^ and a radius of *r*=1 *mm* (as considered in all the above-presented simulation results). This plot demonstrates the high agreement between the results of the proposed model and the experimental results of [3] concerning spatio-temporal evolution and grade association of glioma, even though the proposed model simulates an avascular tumor.

Table 2 depicts the time from the typical detectable size of *r*=1 *cm* to the typical fatal size of *r*=4 cm for all the five different combinations that were investigated in this paper. Additionally, the time intervals for the same *D*/*ρ* pairs are shown in this table for model in [3], which were approximated from the color-scale graph of predicted survival times for a spectrum of in silico patients. It can be observed that almost in all cases, the time that the tumor needs to reach the fatal radius of 4 *cm* according to the proposed model prediction is within the time-intervals of model [3] for the corresponding diffusion-proliferation tumor types.

**Table 2:** Tumor evolution time interval from 1 to 4 cm

**Tumor evolution time**					
***D*(*mm***^**2**^**/*day*)**	***0.005***	***0.3***	***0.005***	***0.3***	***0.04***
***ρ*(/*day*)**	***0.0025***	***0.0025***	***0.04***	***0.04***	***0.015***
*Model in [*[3]	6–9 years	3–18 months	2–3 years	3–6 months	1–2 years
*Proposed Model*	10 years	17 months	2 years	5 months	17 months

Time interval for tumor evolution from the typical detectable size of 1 cm to the typical fatal size of 4 cm.

**Figure 8:**
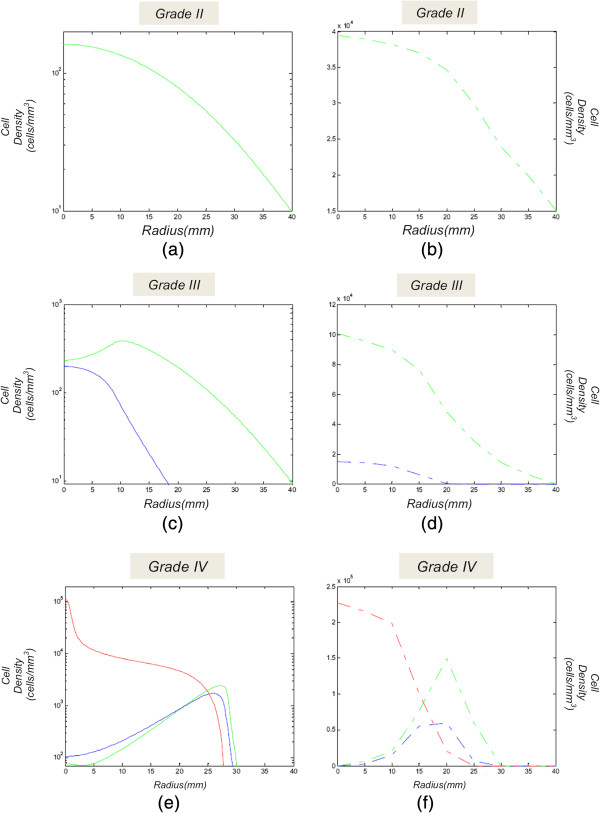
**Glioma grades represented as a plot of cell density for the different cell populations.** Simulations of each grade of glioma represented as a plot of cell density for the different cell population (normoxic, hypoxic and necrotic) with respect to the distance from the center of the tumor. Green curve: normoxic cells, blue curve: hypoxic cells, red curve: necrotic cells. **(a)**, **(c)** and **(e)**: proposed model produced by different diffusion-proliferation pairs, **(b)**, **(d)** and **(f)**: comparable results of [3].

In the model of [3] the simulated T2-detected tumor corresponds to a total cell density greater than the T2-threshold of detection, which according to this paper is approximated to 16% of the maximal tissue-carrying capacity. Following this rationale for comparison purposes, our model is also initialized with a spherical tumor of a density lower than the maximum carrying capacity, yet higher than the minimum detectable value of [3] (0.16·*C*_*m*_<*C*_0_<*C*_*m*_), i.e. *C*_0_=10^5^*cells*/*mm*^3^. Figures 8(a), 8(c) and 8(e) illustrate the simulations of each grade of glioma, represented as a plot of cell density for the different cell populations (normoxic, hypoxic and necrotic), with respect to the distance from the center of the tumor. In Figure 8(e) the hypoglycemic cell population has been included in the normoxic cell population. Comparable curves derived from [3] corresponding to gliomas of similar grades and sizes are shown in Figures 8(b), 8(d) and 8(f). These figures demonstrate that a similar growth pattern is followed in both models, with respect to the different tumor regions in each glioma grade.

The deviations regarding cell-densities between the two models signify their differences and potential limitations. In particular, the proposed model, lacks a consideration of angiogenesis, but considers the impact of glucose gradual reduction on the cell proliferation rate. Moreover, the model of [3] is initialized with a tumor of a tenfold radius, namely the smallest T2 detectable size of *r*=1 *cm*, compared to the proposed model. This large initial size with high cell-density within its entire volume significantly differentiates the final cell densities, i.e. when the tumor has reached its fatal size.

**Figure 9:**
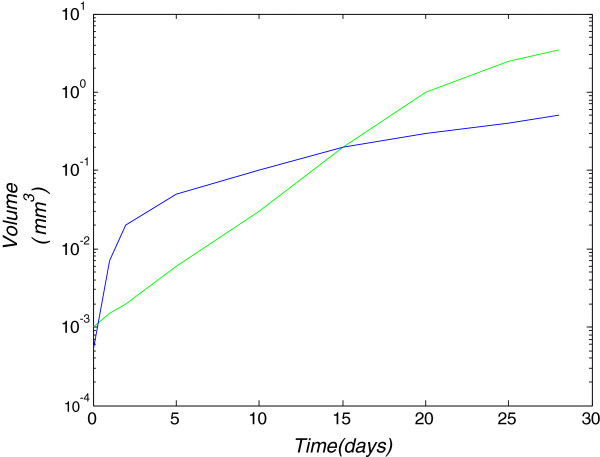
**Tumor volume expansion in respect to time.** Tumor volume expansion in respect to time. Green curve: experimental results of [1], blue curve: proposed model.

**Figure 10:**
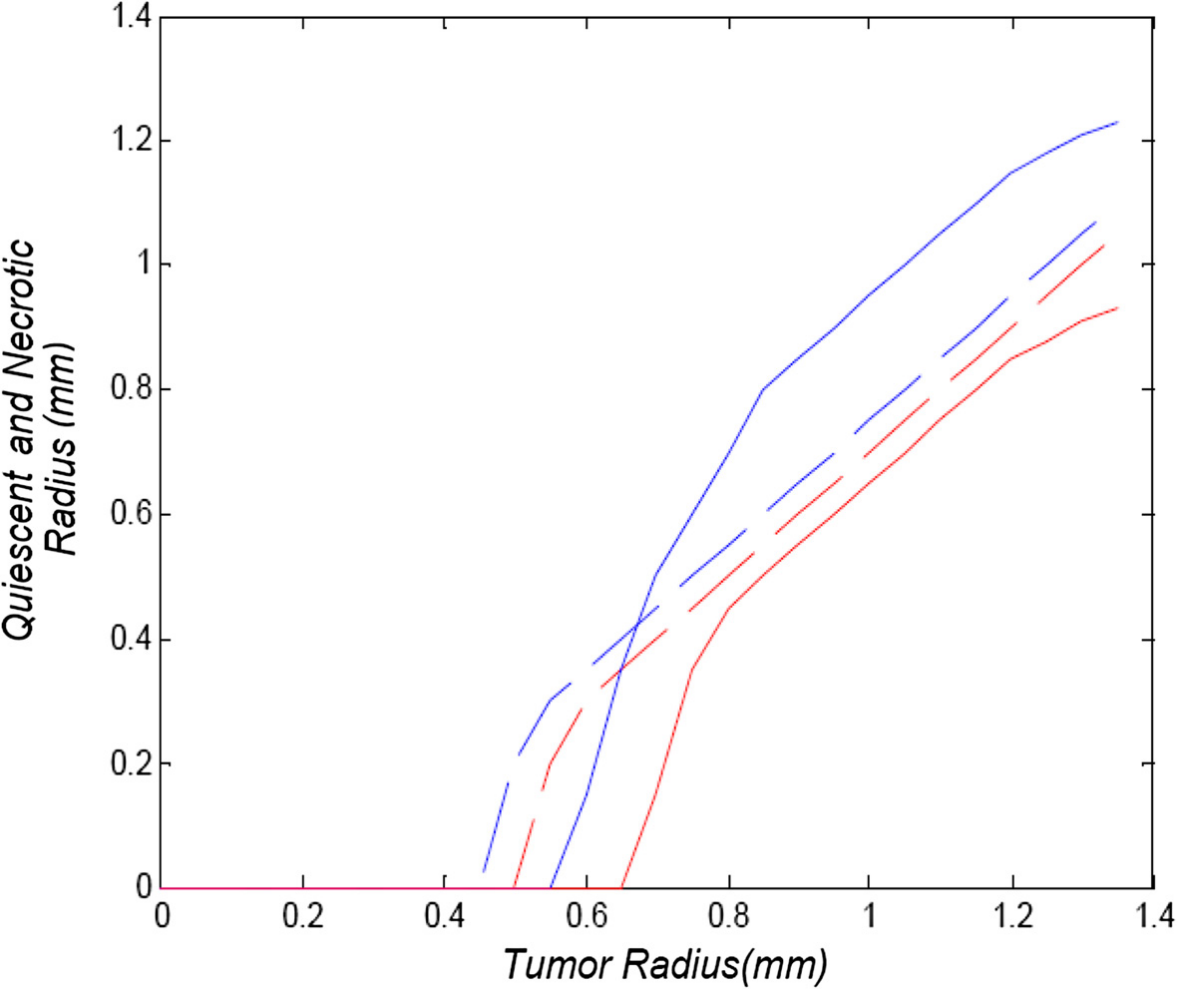
**Quiescent and necrotic radius in respect to different tumour radius.** Quiescent and necrotic radius in respect to different tumour radius. Blue curve: quiescent radius of proposed model, red curve: necrotic radius of proposed model, blue and red dashed curves: corresponding experimental results of [1].

In order to validate the proposed model for an initial tumor of a small radius *r*<1 *mm*, the results are compared to respective outcomes of model [1], which simulates avascular spherical tumor growth based on cell proliferation that depends on the concentrations of oxygen and glucose. Model results are demonstrated for a growing tumor far below its clinical detectable size. The initial concentration values of both nutrients outside the tumor are the same used in our model (*n*_0_=0.28·10^−9^*mol*/*mm*^3^, *gl*_0_=16.5·10^−9^*mol*/*mm*^3^). Since the initial tumor radius here is considered very small, *r*=0.05 *mm*, the concentrations of both nutrient inside the tumor are assumed to have their maximum values, equal to the reference concentration. The model in [1] does not include a diffusion factor for the tumor expansion, but its radius increases corresponding to cell proliferation. For comparison purposes, the diffusion coefficient value of our proposed model has been considered to be proportional to the mean area that each tumor cell occupies. The cell proliferation rate, simulates the tumor expansion due to the increasing space of proliferating cells. Additionally, the proliferation-rate constant in this case has been taken from [1] as *ρ*=0.12/*day*. These modified diffusion and proliferation rates and the tumor initialization for *r*=0.05 *mm*, lead to a similar tumor growth as the one in [1]. The results are presented in Figure 9, where the tumor volume expansion for 28 days in comparison to the respective results of [1] are depicted. Concerning this growth, the radii of quiescent and necrotic regions in respect to the tumor radius are illustrated in Figure 10 and compared to the experimental results of [1]. The quiescent radius corresponds to the hypoxic along with the hypoglycemic region of the proposed model. Figure 10 clearly demonstrates a very good agreement between the experimental data and the simulated growth curves, resulting from our model prediction.

## Discussion and conclusions

Gliomas form an important class of brain cancer with high mortality rate. Mathematical models are increasingly used to understand and predict their behaviour. Identification of the avascular tumor dynamics constitutes the first crucial step towards the investigation of fully vascularized tumors. However, using current modelling techniques one must choose between simulating individual cell behavior and modelling tumors of clinically significant size. Both the continuum and discrete tumor models pose particular limitations. In order to overcome significant restrictions, a multi compartment continuum model appears to be effective in describing how subpopulations of various types of cells proliferate and diffuse, while it is computationally efficient. To this aim, a new continuum three-dimensional spherical mathematical model of avascular glioma growth in an isotropic and homogeneous medium is developed and proposed in this study. This model simulates malignant cell proliferation and invasion behavior, incorporating the interactions between the heterogeneous populations of four different glioma cell phenotypes, namely proliferative, hypoxic, hypoglycemic and necrotic, as well as their tissue microenvironment. Our model is capable of capturing intercellular interactions, spatial cell population heterogeneity and phenotype differentiation. It is validated through multiple simulations in order to determine sensitivity to changes in important model parameters, specifically, the proliferation and cell migration rates [4].

The different cell populations are implemented by means of distinct cellular compartments based on [3], which has been expanded towards the integration of tumor microenvironment with the inclusion of chemical compartments. Unlike the majority of related existing approaches that involve a single nutrient (oxygen) to guide tumor growth, our model incorporates the effects of the concentration changes of two nutrients (oxygen and glucose). The novelty of the proposed model lies in the fact that apart from the combined effect, each nutrient separately affects tumor cell proliferation and viability, while a new cell population is formed due to the lack of glucose, characterizing hypoglycemic cells by a different metabolic profile than hypoxic ones. Moreover, the effects of ECM, MDEs and the two vital nutrients, on cell survival, proliferation and invasion are simulated based on the equations of [32] (for oxygen, ECM and MDEs), which have been extended to include the different production and consumption rates corresponding to the distinct cell types. In essence, our model builds on relevant existing models by combining their capabilities and expanding their exposure and applicability to realistic glioma scenarios. All model variables, e.g. cell densities and concentrations of chemical ingredients are of continuous form described by diffusion-reaction equations. Several simulations have been performed using various diffusion and proliferation rate combinations corresponding to glioma reference values. The model results are presented for different evolution times in order to express the evolutionary characteristics of tumors. Furthermore, the model is validated through comparisons with specific experimental results of glioma models available in the literature, revealing high concordance of our model results with different aspects of glioma spatio-temporal evolution and tumor initialization phases.

Since the clinical cases of substantial significance are above the tumor size of 0.5−1 *cm* radius, our model is initialized with a much smaller tumor-radius *r*=1 *mm* containing only normoxic proliferative cells, as to consider the evolution of tumor composition. Our model predicts tumor radius and composition after its clinical diagnosis, while it also allows tumor initialization at a size far below the detectable in MRI images, so that it can approximate the actual tumor cell-densities since its infancy. As it was shown, the model enables identifying the size of the different cell regions, such as proliferative, hypoxic, hypoglycemic and necrotic, as well as their effect on the overall tumor growth. Therefore, the proposed model may prove useful for determining particular tumor growth parameters, which are hard to obtain from patient data but are crucial in the development of therapeutic strategies for cancer treatment. Moreover, the validation results demonstrate that our model can work effectively and provide the tumor-growth prediction initialized either from a very small or a larger tumor size. This enhances the applicability of the model irrespective of the stage of diagnosis.

In this study the effect of hypoxia as a hallmark of aggressive tumor behavior often met in glioblastomas that has been engaged in the model through nutrients availability/consumption has been associated with cell proliferation and invasion. According to medical experts this is of great significance in clinical practice, since it is associated with resistance to therapy, poorer survival and more malignant tumor phenotypes [2]. Thus, one important utility of models is to simulate a quantitative link between tumor growth kinetics and the hypoxic burden of the tumor. To this aim, our mathematical model describes the spatial and temporal evolution of glioma in terms of concentration of malignant tumor cells. A major strength of this formalism is its potential prospective nature. Although it does not include the prediction of the tumor recurrence location, our method is potentially applicable to orient patient-specific definition of glioma margins.

Furthermore, since our model is able to detect cell densities of even below 1*cell*/*mm*^**3**^, it can effectively characterize the border line regions, i.e. the semi-cancerous regions, as well as the rest extra-tumoral suspicious area, which potentially consist of healthy tissue along with scattered cancer cells. In addition, the area that is occupied by an extra-tumoral edema is likewise considered as suspicious area by the radiologists, since it contains high concentrations of matrix-degradative enzymes (MDEs), which are known to degrade the extracellular material (ECM) and provide a favourable environment to cancer cells in order to migrate. Our model incorporates the variables of both the MDEs and ECM, thus it is able to detect those extra-tumoral suspicious areas containing high MDEs-concentrations and relatively low ECM-concentrations.

Despite its utility and effectiveness, the proposed model in its present form bears certain limitations. Tumor cells with various phenotypes initially develop asymmetric tumor morphologies, since they have different invasion rates and they migrate within an inhomogeneous medium; thus they are non-uniformly distributed. However, they eventually form a circular shape so that the spherical shape of the initial tumor used in our model constitutes a successive real tumor approximation. Furthermore, this model is homogeneous, i.e. it does not take into consideration brain heterogeneity regarding tumor cell invasion. As mentioned in [5], there are several indications that glioma diffuses faster in white than in grey matter. The cell diffusion coefficient considered in the current model is constant throughout the entire medium, while it should vary depending on the invaded tissue area. Along the same direction, model initialization is performed with a virtual tumor that lacks the specific spatial localization of a real tumor surrounded by particular brain anatomic structures (i.e. white and grey matter), where tumor cells would have different invasion rates. Thus, one limitation of the current model is the lack of support for medium heterogeneity, which however can be considered at a further stage of development. Moreover, a real tumor consists of at least three distinct regions of different texture properties (e.g. necrosis, proliferative, edema), where chemicals have different diffusion. The model presented does not consider variable diffusion properties for the nutrients due to intratumoral heterogeneity, since diffusion coefficient values are very difficult to obtain and additionally, such a differentiation would not offer a significant accession to model results.

Cell to ECM interaction (i.e. haptotaxis) and cell to cell adhesion play their specific roles in tumor growth and morphological dynamics. Cell to cell adhesion is important at the early stages of glioma development, while at later stages tumor growth is dominated by cell to ECM and nutrients interactions. Cell to cell adhesion could be incorporated in a macroscopic continuum approach, but has not been included in our model, since it concerns the cellular scale that is not explored in this work. Regarding haptotaxis, it has also been neglected as a distinct diffusion term, but it has been implicitly taken into account through random diffusion of tumor cells towards areas of lower cell concentration, where ECM concentration is higher. Similarly, chemotaxis has been implicitly incorporated through the factor of random diffusion. As another remark, the proposed model does not evaluate the impact of gene expression changes and mutations in cellular behavior, nor does it incorporate the effects of the epidermal growth factor receptor (EGFR) and the associated molecular pathways on tumor growth dynamics.

Overall, the major effort in this work is to demonstrate the results of the changes in the dominant cellular kinetics, i.e. diffusion and proliferation, considering the interactions between tumor cells and their microenvironment. Hence, the current effort forms a comprehensive modelling of the change in the tumor cellular proliferative and invasive phenotype due to the dynamic cell interactions with the microenvironment that contains the vital nutrients. As such, it may miss several key factors that determine cell phenotype, which nevertheless can be readily explored in future models.

Future efforts include the incorporation of several parameters aiming to overcome the limitations. A straight forward direction for model amendment is to address brain tissue heterogeneity and anisotropy issues, in order to take into account the different cell diffusion rates, depending on the invaded anatomic structure (i.e. white or grey matter). This can be easily performed by adopting the topography of imaging modalities (i.e. MRI) similar to other related studies [17]. In the same direction we should consider the model initialization by a real tumor geometry extracted from corresponding medical images. Additionally, primary tumor formation in terms of proliferative, hypoxic and necrotic regions, derived from imaging texture, or biopsy data could also be integrated in this initialization. This primary intratumoral heterogeneity will allow the integration of variable nutrient diffusion properties. Subsequently, the implementation of additional flux factors, such as diffusion due to haptotaxis, chemotaxis and cell to cell adhesion should be addressed. The current model considers the effect of nutrient-concentration changes but it does not take into consideration the increase of waste concentrations, such as lactate and *H*^+^ ion, which act as cell proliferation inhibitors and threaten cell survival. The incorporation of those inhibitors is also considered essential in future developments. Furthermore, the effects of EGFR concentration and related pathways on tumor growth dynamics at a multicellular level will constitute a significant addition. Since the vascular morphology of gliomas has led to the hypothesis that the formation of new blood vessels is essential for tumor growth, future plans will target the investigation of angiogenesis within the proposed model.

According to the medical experts in our team, this model is successful in capturing avascular tumor growth observed clinically. It allows performing patient-specific simulation of different tumor evolution scenarios, towards reliable prognosis of glioma spatio-temporal progression. Since it explicitly incorporates the microenvironment interactions of proliferative, hypoxic and hypoglycemic glioma cells, along with the formation of necrosis, it allows evaluating clinically significant tumor sizes, while it is efficient in describing the dynamics of glioma tumors visualized with medical imaging. This mathematical model provides a means to simulate tumor development scenarios, which may lead to a better understanding of how altering fundamental parameters can influence brain tumor progression; thus it may constitute an important research tool for clinical assessment [50]. In this regard, it can be exploited in cooperation with in-vivo and in-vitro models after establishing the initial diagnosis via a tumor biopsy or surgery. In-vitro invasion assays are important tools for investigating the tumor-matrix interactions and the effects of extracellular macromolecules on these interactions [51-53]. Since such studies are carried out through tightly-controlled experimental conditions, they allow the explicit determination of primary variables, e.g. nutrients initial concentrations [54,55]. On the other hand, in vivo implanted xenographs (human brain tissue) in animal model variations can effectively investigate the reproducible cell migration and the tumor invasion into living non-neoplasmatic brain regions [56]. Thus, both in vitro and in vivo studies of human tumor parts allow the examination of tumor behavior deriving important patient specific parameters, such as the invasion, the proliferation and the nutrients consumption rates [57]. The proposed model can then be initialized, based on these parameters in order to attain a detailed and accurate short- and long-term patient specific prediction.

An important area of model exploration concerns the changes of tumor microenvironment in the context of a specific treatment. The treatment of tumors in the central nervous system represents a formidable challenge, further magnified by the fact that the brain is isolated by the blood brain barrier, rendering the delivery of high doses of chemotherapeutic agents, or gene vectors a very difficult task [58]. This leads to unacceptable toxic systemic levels of drugs. The approaches proposing combinations of local tumor delivery, within the tumor mass and/or surrounding cavity, such as immune-stimulation and cytotoxic gene therapies, appear very promising in achieving the effective treatment of glioblastoma [59]. Additionally, the introduction of new technologies, such as the microchip and convection enhanced drug delivery, will enable the local delivery of treatments, such as drugs and therapeutic gene vectors within the tumor mass, as well as the surrounding area, where the infiltrating tumor cells are localized [60,61]. This provides the motivation for simulating and modelling the targeted cancer treatment scenarios. The multi-compartmental continuous nature of our model enables the integration of therapy-related issues in the form of either a simple factor, or a pharmakokinetic-pharmakodynamic model, which will investigate the beneficial impact of treatment in disease progression.

## Competing interests

The authors declare that they have no competing interests.

## Authors’ contributions

MP conceived the study, designed the model based on existing research efforts, developed its computational implementation, carried out the model simulations and drafted the manuscript. PK and XK participated in the design of the model providing guidelines from the clinical perspective, assisted in parameter estimation process, performed the evaluation of the model results, significantly contributed in the discussion section and suggested the future work extensions. MZ supervised the whole research, contributed to writing and improving the paper, suggested extensions and modifications and revised the manuscript critically. All authors read and approved the final manuscript.

## Supplementary Material

Additional file 1 **Parameter values table.** Additional file 1 contains a multi-page table, which includes the parameter values used for the model simulations. Click here for file

Additional file 2 **Figures of model results concerning intermediate tumor evolution times.** Additional file 2 contains figures of model results (low diffusion-high proliferation glioma), concerning intermediate tumor evolution times, namely 2, 3, 9 and 15 months, as well as the simulation result of the same glioma in the early stage of 1 day after its assumed detection. Click here for file
