# Supplementary material for: Mathematical modelling of spatio-temporal glioma evolution
Source: Theor Biol Med Model. 2013 Jul 24;10:47. doi: 10.1186/1742-4682-10-47 (PMC3734056; doi:10.1186/1742-4682-10-47)
Supplement: Additional file 1 — Parameter values table. Additional file 1 contains a multi-page table, which includes the parameter values used for the model simulations. [file 1742-4682-10-47-S1.pdf]

Table A1 - Parameter values

Parameter values used for the model simulations.

Parameter Values				
Parameters	Symbols	Values	Units	Sources
Tissue maximum carrying capacity	C_m	$2.39*10^5$	$cells/mm^3$	Considered taken into account data of [1]
Initial tumor cell density	C_0	10^4	$cells/mm^3$	Considered taken into account data of [2]
Initial tumor radius	r_0	1	mm	[1]
Diffusion coefficient of proliferative cells	D_c	0.005 - 0.3	mm^2/day	[1]
Diffusion coefficient of hypoxic cells	D_h	D_c*10	mm^2/day	Considered taken into account [1], [3]
Diffusion coefficient of hypoglycemic cells	D_q	D_c	mm^2/day	Considered taken into account [1], [3]
Proliferation rate constant	ρ	0.0025 - 0.04	1/day	[1]
Initial ECM concentration outside the tumor	f_0	10^{-9}	mol/mm^3	[4]
Initial intratumoral ECM concentration	f_{0tumor}	$f_0/10$	mol/mm^3	Considered taken into account data of [4]
Initial MDEs concentration outside the tumor	m_0	0	mol/mm^3	[5]
Initial intratumoral MDEs concentration	m_{0tumor}	$0.5*C_0$	mol/mm^3	[5]
Initial oxygen concentration outside the tumor	n_0	$0.28*10^{-9}$	mol/mm^3	[6], [7], [8]
Initial intratumoral oxygen concentration	n_{0tumor}	n_0*e^{-dr}	mol/mm^3	Calculated from [6]
Initial glucose concentration outside the tumor	gl_0	$16.5*10^{-9}$	mol/mm^3	[6], [7], [8]
Initial intratumoral glucose concentration	gl_{0tumor}	$gl_0*e^{-d(r)}$	mol/mm^3	Calculated from [6]

Diffusion coefficient of MDEs	D_m	0.00864	mm^2/day	[4]
Diffusion coefficient of oxygen	D_n	157.248	mm^2/day	[6], [9]
Diffusion coefficient of glucose	D_{gl}	9.504	mm^2/day	[6], [10]
Oxygen natural decay rate	α_n	0.0375	1/day	Calculated from [4], [11]
Oxygen production rate	β_n	0.5025	1/day	Calculated from [4], [11]
Glucose natural decay rate	α_{gl}	0.11	1/day	Assumed based on [6], [4], [11]
Glucose production rate	β_{gl}	1.1	1/day	Assumed based on [4], [11]
Oxygen consumption rate by proliferative cells	γ_{cn}	$5.2*10^{-12}$	mol/cell*day	Calculated from [6], [7]
Oxygen consumption rate by hypoxic cells	γ_{hn}	$0.2*\gamma_{cn}$	mol/cell*day	Considered taken into account [3], [4], [6], [7], [12]
Oxygen consumption rate by hypoglycemic cells	γ_{qn}	$0.5*\gamma_{cn}$	mol/cell*day	Considered taken into account [4], [6], [7]
Glucose consumption rate by proliferative cells	γ_{cgl}	$1.446*10^{-12}$	mol/cell*day	Calculated from [6], [7], [13]
Glucose consumption rate by hypoxic cells	γ_{hgl}	$10*\gamma_{cgl}$	mol/cell*day	Considered taken into account [4], [6], [13]
Glucose consumption rate by hypoglycemic cells	γ_{qgl}	$0.5*\gamma_{cgl}$	mol/cell*day	Considered taken into account [4], [6], [14]
ECM degradation rate	δ	0.0022388	mm^3/mol*day	Calculated from [4], [11]
MDEs natural decay rate	λ	0.432	1/day	[15]
MDEs production rate by proliferative cells	μ_c	0.006	mol/cell*day	[15]
MDEs production rate by hypoxic cells	μ_h	$2*\mu_c$	mol/cell*day	Considered taken into account [3]
MDEs production rate by hypoglycemic cells	μ_q	μ_c	mol/cell*day	Assumed in this model
Conversion rate of proliferative to hypoxic cells	b_h	$(1- n/n_0)/20$	1/day	Calculated to be proportional to the relative oxygen concentration
Conversion rate of	g_h	0.05	1/day	[1]

hypoxic to proliferative cells				
Conversion rate of hypoxic to necrotic cells due to lack of oxygen	a_h	$b_h/10$	1/day	Considered to be proportional to the proliferative to hypoxic conversion rate
Conversion rate of hypoxic to necrotic cells due lack of glucose	a_{glh}	0.01	1/day	Considered constant in this model
Conversion rate of proliferative to hypoglycemic cells	b_q	$(1- gl/gl_0)/20$	1/day	Calculated to be proportional to the relative glucose concentration
Conversion rate of hypoglycemic to proliferative cells	g_q	0.05	1/day	[1]
Conversion rate of hypoglycemic to necrotic cells due to lack of oxygen	a_q	0.01	1/day	Considered constant in this model
Conversion rate of hypoglycemic to necrotic cells due lack of glucose	a_{glq}	$b_q/10$	1/day	Considered to be proportional to the proliferative to hypoglycemic conversion rate
Conversion rate of proliferative, hypoxic, hypoglycemic to necrotic cells due to contact with necrotic region	a_n	$\log(2)/(50C_m{*}10^2)$	1/day	[1]

References

[1] Swanson KR, Rockne RC, Claridge J, Chaplain MA, Alvord Jr EC, Anderson ARA: **Quantifying the Role of Angiogenesis in Malignant Progression of Gliomas: In Silico Modeling Integrates Imaging and Histology**. *Int Sys Tech: Math Onc Cancer Res* 2011, **71**(24):7366–7375.

[2] Swanson KR, Bridgea C, Murray JD, Alvord EC: **Virtual and real brain tumors: using mathematical modeling to quantify glioma growth and invasion**. *J Neurol Sc* 2003, **216**:1–10.

[3] Martinez-Gonzalez A, Calvo GF, Romasanta LAP, Perez-Garcia VM: **Hypoxic Cell Waves around Necrotic Cores in Glioblastoma: A Biomathematical Model and its Therapeutic Implications**. *Bull Math Biol* 2012, **74**:2875–2896. [World Scientific Publishing Company].

[4] Anderson ARA: **A hybrid mathematical model of solid tumour invasion: the importance of cell adhesion**. *Math Med Biol* 2005, **22**(2):163–186.

[5] Anderson ARA, Chaplain MAJ, Newman EL, Steele RJC, Thompson AM: **Mathematical Modelling of Tumour Invasion and Metastasis**. *J Theor Med* 2000, **2**:129–151.

[6] Kiran KL, Jayachandran D, Lakshminarayanan S: **Mathematical Modelling of Avascular Tumour Growth Based on Diffusion of Nutrients and Its Validation**. *Can J Chem Eng* 2009, **87**:732–740.

[7] Casciari JJ, Sotirchos SV, Sutherland RM: **Mathematical Modelling of Microenvironment and Growth in EMT6/Ro Multicellular Tumour Spheroids**. *Cell Prolif* 1992, **25**:1–22.

[8] Freyer JP, Sutherland RM: **A Reduction in the In Situ Rates of Oxygen and Glucose Consumption of Cells in EMT6/Ro Spheroids During Growth**. *J Cell Physiol* 1985, **124**:516–524.

[9] Mueller-Klieser WF, Sutherland RM: **Oxygen Consumption and Oxygen Diffusion Properties of Multicellular Spheroids from Two Different Cell Lines**. *Adv Exp Med Biol* 1984, **180:**311–321.

[10] Casciari JJ, Sotirchos SV, Sutherland RM: **Glucose Diffusivity in Multicellular Tumor Spheroids**. *Cancer Res* 1988, **48**:3905–3909.

[11] Jeon J, Quaranta V, Cummings PT: **An off-lattice hybrid discrete-continuum model of tumor growth and invasion**. *Biophys J* 2010, **98**:37–47.

[12] Freyer JP, Tustanoff E, Franko AJ, Sutherland RM: **In Situ Oxygen Consumption Rates of Cells in V-79 Multicellular Spheroids During Growth**. *J Cell Physiol* 1984, **118**:53–61.

[13] Gerlee P, Anderson ARA: **A hybrid cellular automaton model of clonal evolution in cancer: The emergence of the glycolytic phenotype**. *J Theor Biol* 2008, **250**:705–722.

[14] Jiang Y, Pjesivac-Grbovic J, Cantrell C, Freyer JP: **A Multiscale Model for Avascular Tumor Growth**. *Biophys J* 2005, **89**:3884–3894. [World Scientific Publishing Company].

[15] Kim Y, Lawler S, Nowicki MO, Chiocca EA, Friedman A: **A mathematical model for pattern formation of glioma cells outside the tumor spheroid core**. *J Theor Biol* 2009, **260**:359–371.
