# Supplementary material for: Mathematical modelling of spatio-temporal glioma evolution
Source: Theor Biol Med Model. 2013 Jul 24;10:47. doi: 10.1186/1742-4682-10-47 (PMC3734056; doi:10.1186/1742-4682-10-47)

Figure 1A - Model simulation of a low diffusion-high proliferation tumor for 1 day

Model simulation of a low diffusion / high proliferation tumor for 1 day: (a) cell density with respect to the tumor radius, (b) tumor cells dispersal in a 2D section, (c) oxygen and (d) glucose concentrations with respect to the tumor radius. In this early stage, it is demonstrated that the tumor radius has increased to approximately r=1.6mm and all cancer cells remain proliferative, namely no hypoxia, and/or hypoglycemia have appeared yet. This is verified by Figures 1A(c) and 1A(d) where oxygen and glucose concentrations with respect to the tumor radius are illustrated. It is clear that even in the central part of the tumor both nutrients are still adequate.

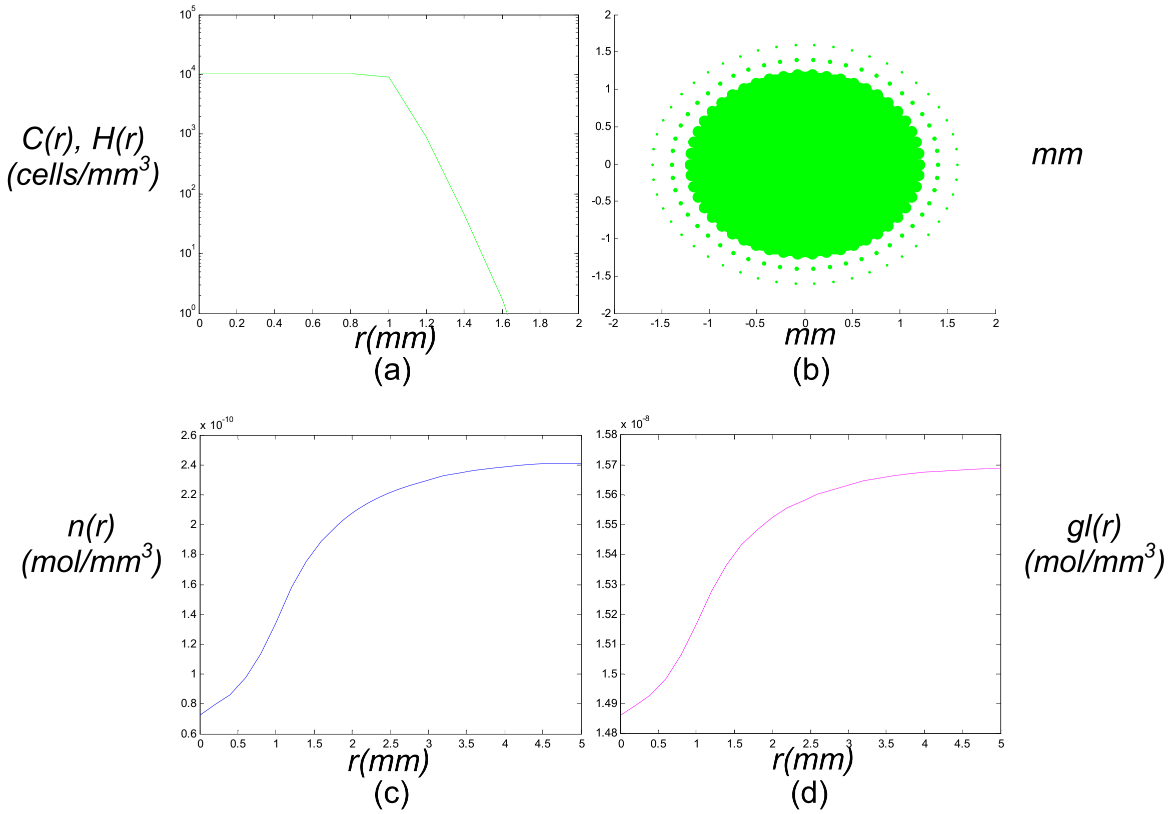

Figure 2A - Model simulation of a low diffusion-high proliferation tumor for 60 days (2 months)

Model simulation of a low diffusion / high proliferation tumor for 60 days (2 months): (a) cell density with respect to the tumor radius, (b) tumor cells dispersal in a 2D section, (c) oxygen and (d) glucose concentrations with respect to the tumor radius. These simulation results demonstrate that the hypoxic cell density have increased at the tumor center and a hypoglycemic cell population has appeared, due to the glucose inadequacy, shown in Figure 2A(d). Moreover, note that the hypoxic radius is slightly larger than the normoxic one. This result can be explained by the consideration that hypoxic cells are more invasive, i.e. they have higher diffusion coefficient and hence at the particular time point they have migrated further than the normoxic cells. However, at a later time, they will convert back to normoxic, since they have moved to a fully oxygenated area. Apart from hypoxic and hypoglycemic cell populations a very small necrotic area has made its appearance in the tumor core, where hypoglycemia and hypoxia coexist, due to the lack of both nutrients, where oxygen exhibits a more extensive shortage (Figure 2A(c)). Furthermore, it can be observed that even at the tumor center, the concentration of proliferative cells is still higher compared to all the other densities.

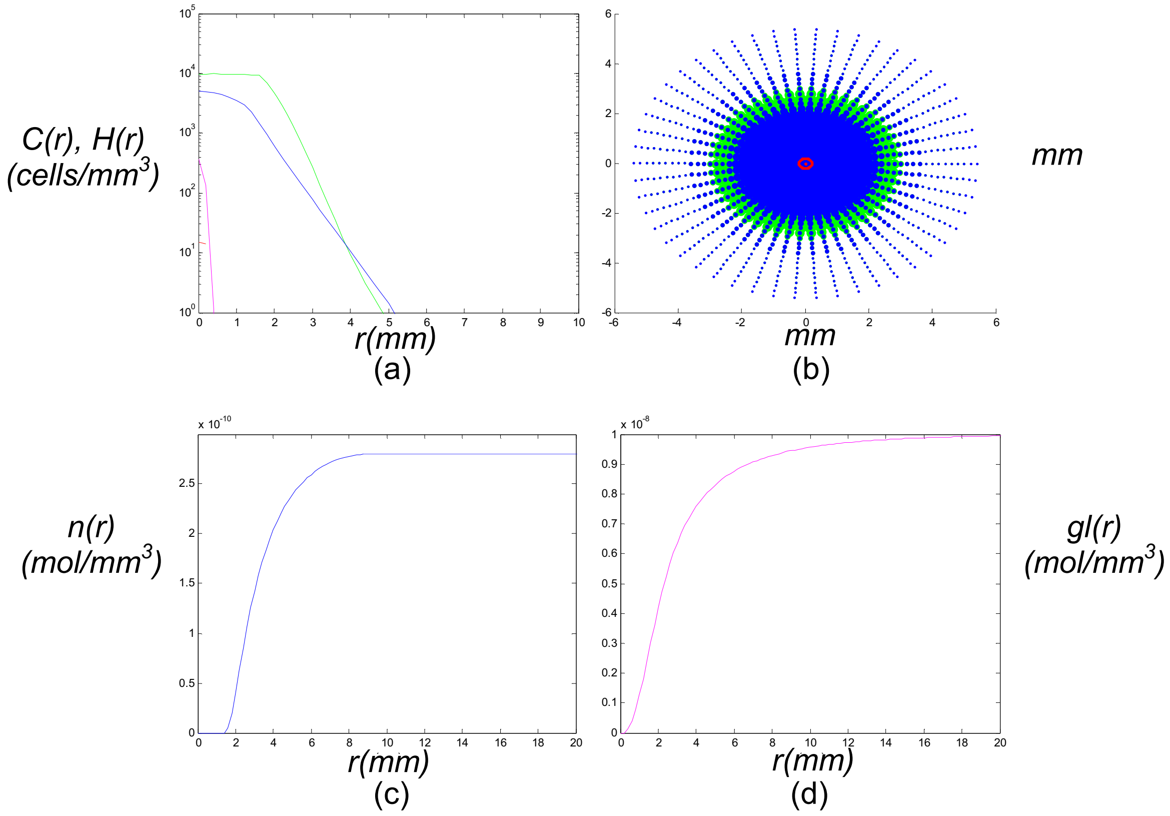

Figure 3A - Model simulation of a low diffusion-high proliferation tumor for 90 days (3 months)

Model simulation of a low diffusion / high proliferation tumor for 90 days (3 months): (a) cell density with respect to the tumor radius, (b) tumor cells dispersal in a 2D section, (c) oxygen and (d) glucose concentrations with respect to the tumor radius. After 3 months simulation, the hypoxic and hypoglycemic areas (blue and magenta curves respectively in Figure 3A(a)) have grown, while they constitute the majority of cell populations in the tumor core. The necrotic density (red curve and area in Figures 3A(a), 3A(b)) has also increased in the core and extends up to the radius of r=2mm. The hypoxic, hypoglycemic and necrotic populations have increased at the expense of proliferating cells (green curve in Figure 3A(a)), which now constitute the minority for the tumor radius of r<1mm.

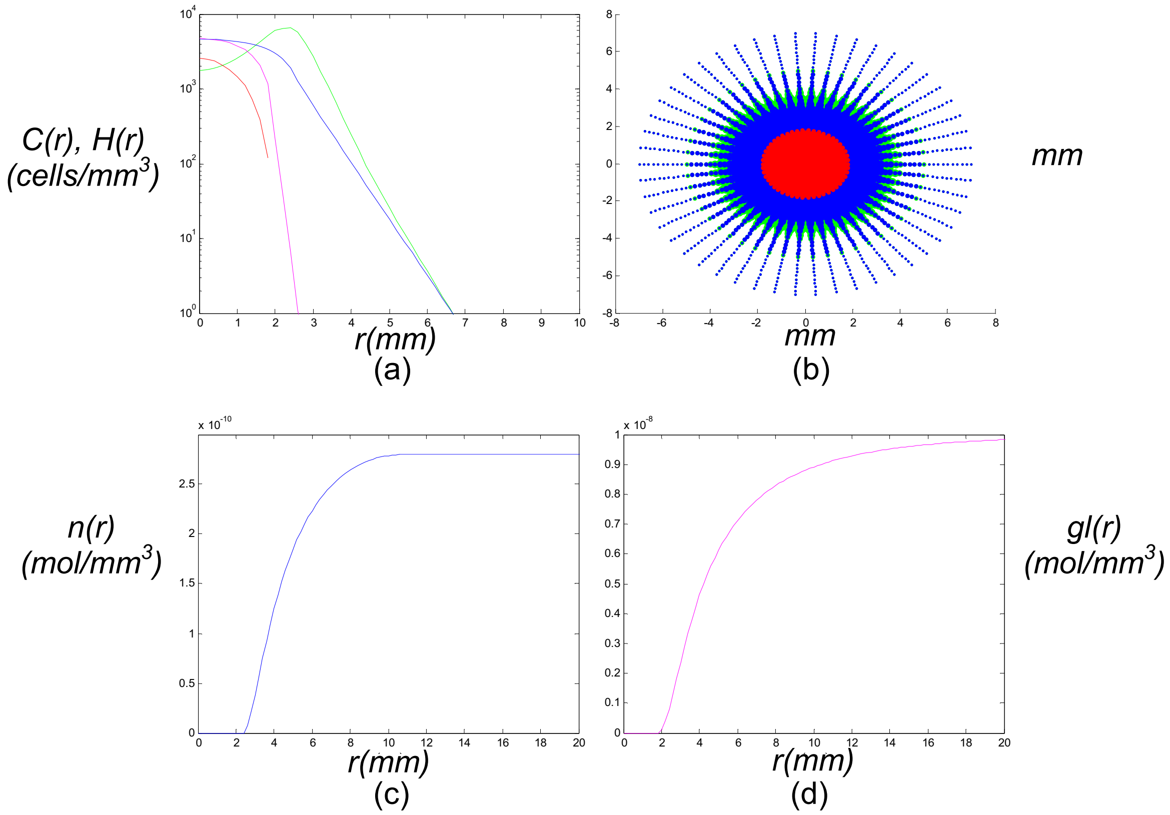

Figure 4A - Model simulation of a low diffusion-high proliferation tumor for 270 days (9 months)

Model simulation of a low diffusion / high proliferation tumor for 270 days (9 months): (a) cell density with respect to the tumor radius, (b) tumor cells dispersal in a 2D section, (c) oxygen and (d) glucose concentrations with respect to the tumor radius. After 9 months the separate zones become more distinct, since the cells that compose each prevail on all the others. It can be clearly observed that proliferating cells no longer exist in the central part of the tumor.

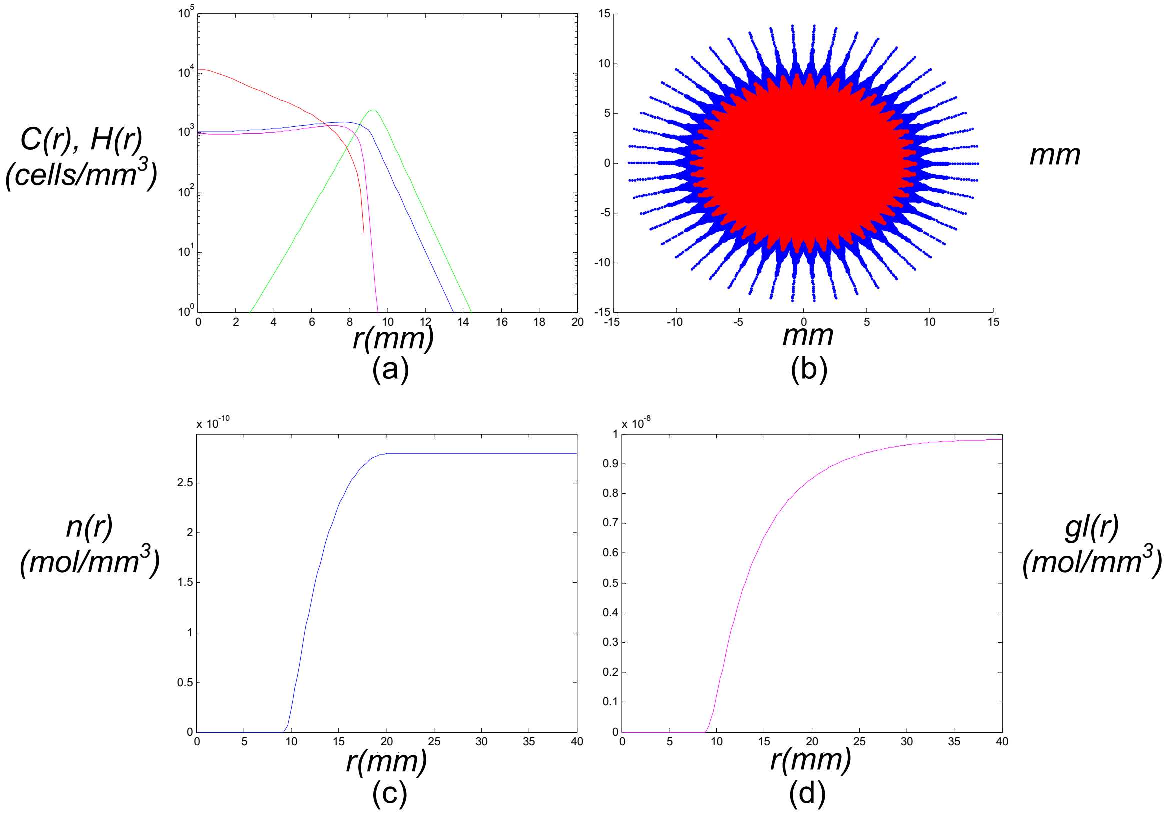

Figure 5A - Model simulation of a low diffusion-high proliferation tumor for 450 days (15 months)

Model simulation of a low diffusion / high proliferation tumor for 450 days (15 months): (a) cell density with respect to the tumor radius, (b) tumor cells dispersal in a 2D section, (c) oxygen and (d) glucose concentrations with respect to the tumor radius. After 15 months, the formation of the extended necrotic core, surrounded by the hypoglycemic and hypoxic regions and the outward proliferating zone are demonstrated.

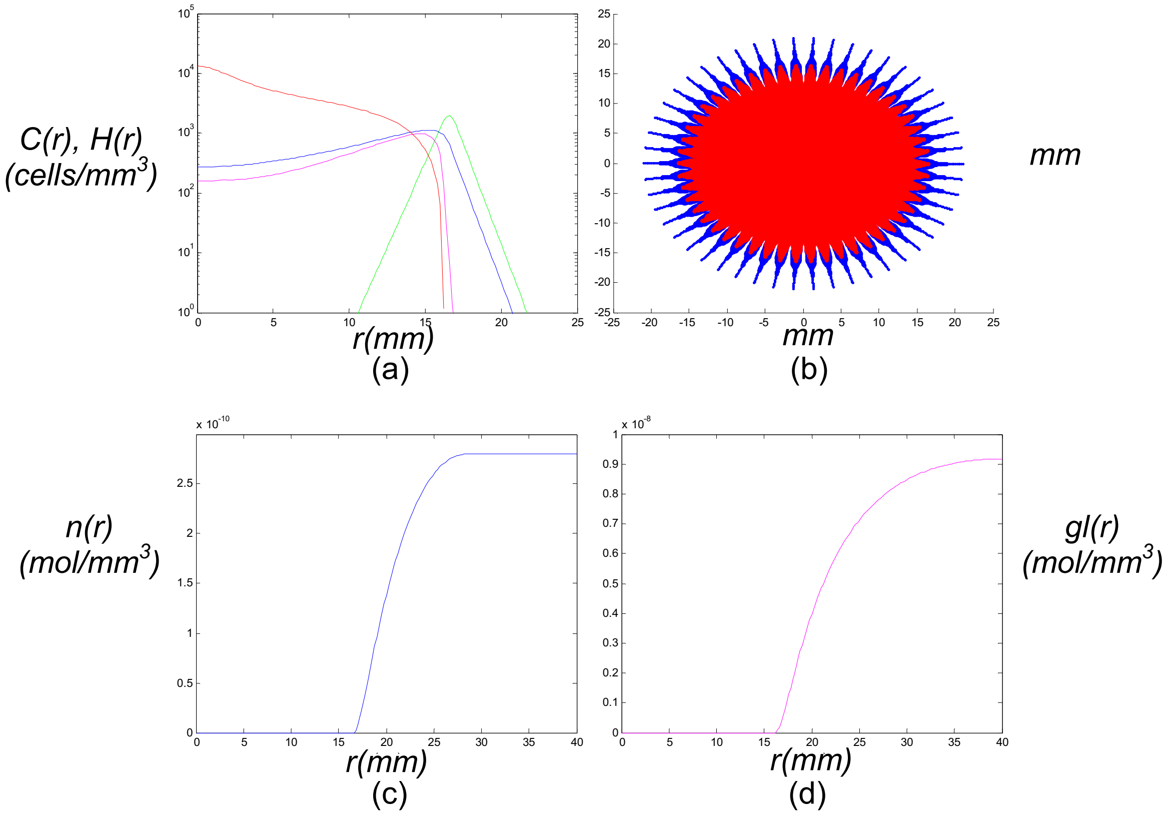

Supplement: Additional file 2 — Figures of model results concerning intermediate tumor evolution times. Additional file 2 contains figures of model results (low diffusion-high proliferation glioma), concerning intermediate tumor evolution times, namely 2, 3, 9 and 15 months, as well as the simulation result of the same glioma in the early stage of 1 day after its assumed detection. [file 1742-4682-10-47-S2.pdf]
